# Diverse Applications of Electronic-Nose Technologies in Agriculture and Forestry

**DOI:** 10.3390/s130202295

**Published:** 2013-02-08

**Authors:** Alphus D. Wilson

**Affiliations:** USDA Forest Service, Southern Research Station, Center for Bottomland Hardwoods Research, Southern Hardwoods Laboratory, P.O. Box 227, Stoneville, MS 38776, USA; E-Mail: dwilson02@fs.fed.us; Tel.: +1-662-686-3180; Fax: +1-662-686-3195

**Keywords:** artificial olfaction, electronic aroma detection, volatile organic compounds

## Abstract

Electronic-nose (e-nose) instruments, derived from numerous types of aroma-sensor technologies, have been developed for a diversity of applications in the broad fields of agriculture and forestry. Recent advances in e-nose technologies within the plant sciences, including improvements in gas-sensor designs, innovations in data analysis and pattern-recognition algorithms, and progress in material science and systems integration methods, have led to significant benefits to both industries. Electronic noses have been used in a variety of commercial agricultural-related industries, including the agricultural sectors of agronomy, biochemical processing, botany, cell culture, plant cultivar selections, environmental monitoring, horticulture, pesticide detection, plant physiology and pathology. Applications in forestry include uses in chemotaxonomy, log tracking, wood and paper processing, forest management, forest health protection, and waste management. These aroma-detection applications have improved plant-based product attributes, quality, uniformity, and consistency in ways that have increased the efficiency and effectiveness of production and manufacturing processes. This paper provides a comprehensive review and summary of a broad range of electronic-nose technologies and applications, developed specifically for the agriculture and forestry industries over the past thirty years, which have offered solutions that have greatly improved worldwide agricultural and agroforestry production systems.

## 1. Introduction

A wide variety of sensor technologies are utilized in modern agriculture and forestry to obtain accurate information on crop, soil, weather, and environmental conditions. Sensing tools are used in these industries for a multitude of applications in the manufacturing of agricultural and forest products, particularly for quality control and monitoring industrial processes. Agricultural and forestry management methods strongly rely on a spectrum of sensor technologies ranging from aerial remote sensing, portable field weather stations, greenhouse environmental sensors, electrochemical sensors, electronic noses, biosensors, and sophisticated wireless sensor networks [1]. Electronic-nose devices are being used with increasing frequency because they allow the acquisition of real-time information about the chemical and physical nature and quality of plants, plant and animal products, and gas effluents released from agricultural and forestry products throughout the entire food and fiber production cycle. The continuous-monitoring capability of e-nose devices provides a means of assuring that production methods and outcomes meet quality specifications (standards) and demands required by regulatory agencies and the consumer for ultimate salability in commercial markets.

The invention of diverse electronic nose (e-nose) sensor types and instruments, based on different electronic aroma detection (EAD) principles and mechanisms, has led to the development of e-nose applications for diverse disciplines within the plant sciences [2]. Gas sensing-applications utilizing e-nose devices in agriculture and forestry are naturally divided into two major groups, including those developed for commercial and industrial applications of products derived from: (1) small nonwoody (herbaceous) plants, used as agronomic crop (food) plants, and animals within the agricultural industry, and from (2) larger woody plants used as ornamentals, landscape structure, fiber, or wood production within the forestry industry. Thus, the agriculture and forestry industries handle the majority of plant and plant-derived products that originate from wild and domesticated plant species throughout the world. The industrial sectors comprising each of these two plant product-associated industries are vast due to the large number of plant species and product types that are exploited by world commerce. Animal-derived products in agricultural are primarily derived from the commercial meat-producing industries including livestock, fish, poultry, and various milk-derived products.

Plants, as a taxonomic group, collectively synthesize a very large range of organic (carbon-based) compounds that are categorized into many different chemical classes. These diverse organic chemicals are produced as a result of biochemical or metabolic processes that take place within specialized cells of many different types of differentiated plant tissues in the root, stems, and leaves. Leaf tissues are particularly rich in diverse organic compounds as a consequence of being the chief organ that captures solar energy in the form of radiation and stores that energy as chemical energy, required for all cellular processes and biosynthetic pathways that produce the myriad of organic compounds present within plants. Some chemical monomeric compounds are linked together to form various types of structural or functional biopolymers such as carbohydrates, lipids, proteins, and nucleic acids. These polymeric compounds generally have low volatility as a result of their high molecular weight. Other smaller intermediates of biochemical processes are modified to form a variety of primary and secondary metabolites performing many cellular or biochemical functions. Relatively small molecular weight organic compounds, generally <350 Daltons [3], may contain various polar and nonpolar functional groups that contribute to volatility. Compounds having high vapor pressure (low boiling point), called volatile organic compounds (VOCs), are particularly conducive to e-nose detection because they are easily vaporized (made airborne as gases), greatly increasing their accessibility for detection within sampled air.

The detection of plant- or animal-derived VOCs using electronic-nose devices usually is performed on simple to complex mixtures of volatilized organic compounds derived from living tissues or from nonliving processed products derived from plant or animal cells. The most common purpose of such analyses with e-nose instruments is to identify the source (plant, animal, or derived product) that produced the unique mixture of organic compounds present in the sample analyte, not the individual compounds present in the sample mixture. A second common purpose for performing e-nose analyses is to assess one or more chemical, biological or physical characteristics about the source that released the sample analytes. Characterizing the source of a sample may be done for the specific purposes of determining product consistency, quality, purity, age, or state of merchantability. For example, e-noses are used to evaluate fruit freshness, ripeness, and shelf-life. For commercial wines, the bouche from different bottles of a wine batch or vintage may be analyzed for uniformity, fruitiness, aroma, age, and other characteristics that determine quality, merchantability, and appropriate price in the market place.

The agriculture and forestry industries have become highly dependent upon electronic-nose devices because of the capability of these instruments to recognize the presence of specific gas mixtures that are produced or released during or as a consequence (byproduct) of various manufacturing processes. The aroma characteristics of agricultural products, particularly in the food industry, contribute immensely to product value and appeal to consumers and thus often determine the salability of manufactured goods. For these reasons, quality control (QC) of the aroma characteristics of manufactured products is of paramount importance because product consistency is essential for maintaining consumer brand recognition and satisfaction [4]. Other common QC manufacturing applications of e-noses are in product grading, uniformity, mechanical processing controls, and monitoring environmental effluents released from manufacturing processes.

The purpose of this review is to provide a thorough overview of the diversity of uses for electronic-nose technologies within the wide spectrum of applications in the agricultural and forestry sectors and to provide numerous examples demonstrating the many ways in which e-nose devices have improved the quality and efficiency of food and fiber production processes within these industries.

## 2. The Nature of Electronic-Nose Devices and Target Chemicals Detected

Electronic-nose devices are different from most other instruments used in chemical analyses in that they are mainly designed to recognize gas mixtures as a whole without identifying individual chemical species within the mixture. For this reason, e-noses generally are not primarily utilized to determine the entire composition of complex gas mixtures, but rather are most useful for determining the sources (from which gas mixtures were derived), the identity of specific gases present, and associated physicochemical characteristics. The sources of gas analytes may be either natural or synthetic organic sources that produce VOCs or inorganic gas sources releasing various types of volatile inorganic compounds (VICs) as gases. In fact, e-noses are commonly used to detect both natural and manmade organic and inorganic pollutants in the environment [5]. All of these categories of volatile gases are produced in association with many different agricultural and forest-product industrial sectors.

The types and mixtures of VOC gases detectable by e-nose instruments depend on the sources, uses, and nature of the products being manufactured in individual agricultural and forest-product industries. Some major categories and common sources of VOCs detected by e-noses are presented in Table 1.

A large diversity of agricultural and forest-products industry waste byproducts are produced in association with plant harvesting, product manufacturing, and associated industrial processes. Many of these manufacturing waste byproducts are either hazardous to human health or are olfactorily offensive, requiring the use of e-nose type sensors to continually monitor effluent levels being released into air soil, and water resources from industrial processes [5]. Some offensive agricultural waste effluents monitored by e-nose devices are listed, along with human olfactory detection and recognition thresholds, in Table 2. Many of these compounds also are produced as a result of microbial or chemical degradation of raw or processed agricultural or forest-products, before or after harvesting, during the manufacturing process, or in storage before or after processing.

Generally, the concentration levels required for human olfactory detection are significantly lower than the concentrations required for recognition. Detection of these compounds released from tainted products usually indicates that these commercial products have undergone microbial degradation to produce staling metabolic products and therefore must be culled because they no longer have merchantable value. Thus, e-nose sensors in this case serve to maintain quality control of agricultural products for human safety and to preserve or avoid contamination of other perishable goods or products that may be in close proximity or contact with spoiled products.

There are two major sources of VOCs, emitted into the atmosphere as a result of agricultural and forestry-product industrial processes, that are detectable with e-nose devices. Biologically-generated VOCs account for the majority of carbon released in the form of VOCs by plants and animals in agricultural crop fields, grazing lands, natural forests and plantations or tree farms. The major sources of biologically-generated VOCs include methane from livestock, wetlands, and agricultural fields (about 340 teragrams of carbon per year); and also isoprene (C_5_H_8_) and isoprenoid or terpenoid (C_5_H_8_)_n_-compounds released from plants (mostly from leaves), accounting for an estimated total of 1,150 teragrams of carbon per year in the form of VOCs [6]. Anthropogenic sources, derived from harvesting and manufacturing activities from various industries, account for the remainder of VOCs emissions, totaling about 140 teragrams of carbon released per year in the form of VOCs such as hydrocarbon solvents, fuels, cleaning products, refrigerants, pesticides, and gaseous or volatile liquid industrial byproducts (wastes) [6].

Volatile inorganic compounds (VICs) also are a significant pollution-emission problem arising from industrial activities related to agriculture and forestry production systems such as the industrial production of pesticides, fertilizers, and other chemicals needed in agroforestry production. Similarly, VICs may be detected by a range of different e-nose devices that are commonly used in the detection, monitoring, and control of environmental pollution because VICs are common chemical pollutants [5]. Some of the more common VIC pollutants released as gas effluents from agroforestry production systems include CO, CO_2_, NH_3_, NO_2_, NO_x_, H_2_S, SO_2_, as well as heavy metals (e.g., arsenic, cadmium, lead, mercury, and zinc) released into agricultural systems via fertilizers, organic wastes such as manures, and in industrial waste byproducts.

### 2.1. Electronic Nose Types and Characteristics

The diversity of EAD technologies utilized in electronic-nose devices include a variety of different sensor types that operate based on different gas-sensing principles, ranging from bulk acoustic wave (BAW), calorimetric or catalytic bead (CB), carbon black composite (CBC), catalytic field-effect (CFET), conducting polymers (CP), complementary metal oxide semiconductor (CMOS), electrochemical (EC), fluorescence (FL), metal oxide semiconductor (MOS), Metal oxide semiconductor field effect transistor (MOSFET), micro-electromechanical systems (MEMS), quartz crystal microbalance (QCM), optical fiber live cell (OF-LC), and surface acoustic wave (SAW) gas sensors. Some advantages and disadvantages of these various e-nose sensor types have been summarized previously [4], although the utility of individual sensors largely depends on the particular application, environmental conditions, and types of gas analytes to be detected.

A complete electronic-nose system typically consists of several integrated and/or interfaced components including a multisensor array (composed of several to many gas sensors with broad sensitivity and cross-reactivity or partially-overlapping selectivity), a data-processing and analysis unit such as an artificial neural network (ANN), software having digital pattern-recognition algorithms, and often aroma reference-library databases containing stored files with digital fingerprints of specific aroma reference (signature) patterns [2,4]. Broad spectrum cross-reactive sensor arrays usually are composed of incrementally-different sensors chosen to respond to a wide range of chemical classes and capable of discriminating diverse mixtures of possible analytes that may be detected. Narrow-spectrum sensor arrays are designed for application-specific e-noses to detect a limited range of analytes from specific chemical classes known to be the only analytes of interest for detection. The electronic outputs, derived from all responses of the individual sensors in the sensor array, are converted into digital values by a transducer and assembled together to produce a distinct electronic aroma signature pattern (EASP) that is determined by the collective sensor-array responses to the entire mixture of VOC or VIC gas analytes present in the sample being analyzed. Identification and classification of the analyte mixture is accomplished through recognition of this unique aroma signature (electronic fingerprint) from comparisons with the reference databases in a library of known EASPs—much like similar libraries used in gas chromatography-mass spectroscopy (GC-MS) analyses. The reference library of aroma signature patterns for known samples is constructed prior to analysis of unknowns and is used to form the recognition files used by pattern-recognition algorithms to arrive at a percentage match value with known patterns in the library. Sensory output patterns derived from analytes that do not match any patterns of known gas mixtures to a significant level (>90%) are determined to be unidentified or unknown. Therefore, false-positive determinations are usually rare when analyte samples are from a known sample type (source), fully represented (variation accounted for) in the reference library, and confidence-level controls are set appropriately to make effective discriminations.

### 2.2. Considerations of E-Nose Designs for Specific Applications

The suitability of an electronic nose for a specific application is highly dependent on the required operating conditions (environment) of the sensors in the array and the composition of the target analyte gases being detected. A proper selection of an appropriate e-nose system for a particular application must involve an evaluation of systems on a case-by-case basis. Some key considerations involved in e-nose selection for a particular application must necessarily include assessments of the selectivity and sensitivity range of individual sensor arrays for particular target analyte gases (likely present in samples to be analyzed), the number of unnecessary (redundancy) sensors with similar sensitivities, as well as sensor accuracy, reproducibility (preciseness), response speed, recovery rate, robustness, and overall performance.

The effective design of electronic-nose devices for agricultural and forestry applications depends on several factors including the specific gas-sensing application(s) to be employed, the range of target analyte chemicals to be detected, the required operating conditions (environment) of the instrument, the selectivity and sensitivity ranges for detection required, and various operational requirements such as run speed and cycling time between samples, sensor array recovery time, data analysis and result-interpretation requirements [4]. In the recent history of e-nose sensor design, it has become apparent to some design engineers that there are many advantages to designing electronic-nose devices based upon the specific application(s) for which the instrument will be applied, instead of basing the design on a more generalized goal of producing a versatile instrument with a broad-range of gas-sensing capabilities and applications. Logically, it would appear to e-nose manufacturers that a more general e-nose device would have wider applications and could be sold to clients in many different industries. In reality, the needs and specification requirements of individual industries are so vastly different and specific that a generalist-type instrument is often unusable due to the inflexibility of operating parameters, detection limits, and sensing capabilities and requirements (*i.e.*, specific types and range of analytes that must be detectable with the instrument). From these experiences, it has become apparent that application-specific e-noses serve individual customers or industries to greater levels of satisfaction because such instruments do a better job of detecting the specific analytes required and can be designed to produce results (instrument outputs) in customized formats that are most useful for data analysis and use by specific narrow industries. Thus, narrow-spectrum sensor arrays designed for application-specific e-noses often are considerably cheaper because the number of sensors required in the array for effective discriminations is significantly reduced.

Sensor array selectivity for specific target VOCs is a major factor for consideration in designing e-nose devices or in selecting specific sensor types to include in the array for a particular gas-sensing task. For example, MOS sensors are particularly useful for monitoring VOCs due to such advantages as low cost, rapid sensor response and recovery times, and ease of e-nose manufacture [7–11]. However, certain MOS sensors are not widely used for interior environmental-monitoring applications, such as monitoring indoor air quality in buildings, because they are often limited by the lack of selectivity towards VOCs from similar chemical classes. This difficulty in distinguishing between related VOC species results from similar elemental composition (primarily carbon and hydrogen) in molecular structure. Thus, pollutants consisting of such VOCs as benzene, formaldehyde, toluene and xylene that cause indoor environmental illnesses (building-related sicknesses) often cannot be distinguished without improving sensor selectivity to discriminate between structurally-similar VOCs [12]. Wen and Tian-mo [13] proposed the use of a mixed-oxide MOS sensor consisting of SnO_2_-TiO2 doped with silver (Ag) ions to improve selectivity for VOC detection. They found this mixed-oxide sensor exhibited differential selectivity to different VOCs which varied at different operating temperatures. Furthermore, quantum chemistry calculations showed that differences in orbital energy of structurally-different VOC molecules may be a qualitative factor that affects the selectivity of mixed-oxide MOS sensors.

Sensor selection for individual e-nose systems is of paramount importance in order to achieve effective and efficient aroma identifications or classifications. A fundamental design concept for an array of sensors used in electronic noses is that each sensor should maximize overall instrument sensitivity and provide different selectivity profiles over the range of target-gas analytes to be detected or classified for a particular application [14]. Ideally, a sensor array should consist of individual sensors that produce a different response to a given odor analyte so that a unique aroma pattern is created. If there is difficulty in obtaining unique aroma patterns for different gas analytes, sensor selection must be modified or the number of sensors adjusted when classification, performance, cost, or technological limitations are issues of concern.

The first step in sensor selection and adjustments within the sensor array is to analyze the sensor's output and performance to a range of target gas analytes to be detected and determine whether there is any redundancy (cross-sensitivity) or irrelevancy (lack of sensitivity) of individual sensors that reduces the effectiveness of analyte discriminations [14]. Inappropriate sensor selection or a poor sensor array configuration can result in the deterioration of e-nose performance. One major advantage of e-nose devices is the large number of sensor types that are available for inclusion in a sensor array of different e-nose types and for different gas-sensing applications. Large libraries of sensor types are available for selection in many cases to facilitate the custom design of an e-nose for detecting specific target analytes [4]. The development of mobile portable e-nose devices usually involves a reduction in sensor number (relative to larger bench-top laboratory instrument versions) and more precise selection of specific sensor types in the array to optimize performance for specific applications and minimize size and costs.

Electronic nose sensor designs frequently are inspired by biological olfactory systems that are analyzed and modeled, serving as a basis for designing e-noses by mimicking the functionality of these natural systems to produce so-called biologically-inspired (biomimetic) e-nose devices. In reality, e-nose instruments neither truly mimic the mechanical structure nor functionality of biological olfactory systems due to their complexity and huge sensor diversity, e.g., more than 300 human olfactory binding proteins (OBP) have been identified in the human olfactory system. Nevertheless, Che Harun *et al.* [15] have developed an improved concept for an electronic nose that combines three large chemosensor arrays (300 resistive elements per array) with two micro-packages, each containing a column inspired by the study of the human olfactory mucosa and nasal cavity, that significantly enhances the ability of the e-nose to discriminate complex odors. Further studies of biological olfactory receptors (ORs), consisting of a large family of G-protein coupled receptor proteins (GPCRs) responsible for sensing the ambient chemical environment [16,17], will no doubt result in future e-nose sensor designs that take into account the 3-dimensional structural confirmation of odorant molecules to produce e-nose devices with greater discrimination capabilities than is currently achieved based only on the electronic effects of odorants as they adsorb to the surface of contemporary e-nose sensors.

The relationship between the properties of odorant molecules (structural conformation and composition) and the resulting odors or aromas recognized by biological olfactory systems provides a means of measuring or quantifying odors and placing them into categories based on measured likenesses or differences in olfactory characteristics. Likewise, attempts to quantify aroma properties of different classes of VOCs using sensory outputs from electronic noses have provided ways of categorizing aromas using various electronic metrics. This process generally is accomplished using data-manipulation algorithms, such as artificial neural network (ANN) systems, that look for differences between aromas based on selected measurable parameters.

Odorant molecular recognition in biological systems involves binding of odorant molecules to olfactory-receptor sites with either attractive or repulsive (electrostatic) chemical interactions that can be associated with the presence of odotopes (exposed charges of specific shapes, types and numbers resulting from fragments of molecular shape [18]) present on odorant molecules. These electrostatic interactions can occur between fixed charges, dipoles, induced dipoles or atoms able to form weak electron bonds (e.g., hydrogen bonds); and include repulsive interactions (electrostatic or quantum-mechanical electron-shell exchange repulsion) as well as attractive forces between odorants and receptors. Every possible change in molecular structure of odorants alters the set of exposed surface features (odotopes) capable of forming such attractive or repulsive interactions, and thus is affected by molecular shape and charge distribution.

Odotope theory suggests that the smell of a molecule is due to the pattern of excitation that results from the interaction of exposed atoms or functional groups in odorant molecules to specific types and numbers of excitable sensory receptors to which they bind [19]. This theory accounts for the sensing of a considerable number of possible smells based on the many permutations of interactions between odorant odotopes and different types of sensory-receptor binding sites. Even if one assumes that sensor receptors are only on or off (binary), this scheme potentially accounts for considerable combinations of possible sensory input to discriminate odor types depending on the number of atoms, odotopes and receptor types involved in these interactions. Combining multiple odotopes of odorant molecules with possible variable intensity of excitation for each receptor would enable such as a system to detect and discriminate a vast number of possible odorants. If the large number of odorant receptor types (binding sites) represent sensory analogs of odotope categories, then the possibilities for sensory discrimination of different VOCs becomes astronomical [18].

Good empirical evidence to support the odotope theory is the ability of humans to detect the presence of functional groups with excellent reliability. Examples include the case of thiols (–SH) that impart the familiar sulphur smell to compounds, nitriles (–C≡N) that yield a metallic character to any smell, isonitriles (–N≡C) with an unpleasant, flat metallic smell, oximes (–C=NOH) with a green-camphoraceous odor, nitro groups (–NO_2_) with a sweet-ethereal character, and low molecular weight aldehydes (–C=O(H)) with a rotten-fruit smell [18,20]. Humans can, in some cases, even recognize the presence of specific bond types between atoms in an odorant. The acetylenic triple bond between carbon atoms (–C≡C–) in alkyne hydrocarbons imparts a mustard-like smell to molecules [18]. However, exceptions do exist such as compounds having very similar chemical structure but dramatically different odors, and compounds with completely different structures having similar odors [21]. Apparently, other unknown factors are involved in odorant characterization and recognition by the human brain based on sensory input derived from odorant-sensor (olfactory receptor protein) interactions.

Odorant molecules generally must be volatile, hydrophobic, and have a molecular weight less than 300 Daltons to be detectable by olfactory systems. The size requirement appears to be a biological constraint related to sensory-receptor size-response limitations. Vapor pressure (volatility) falls rapidly with molecular size, but does not explain why larger molecules have no smell given that some of the strongest odorants (e.g., some steroids) are large molecules. A further indication that the size limit of odorants is related to the chemoreception mechanism is that specific anosmia (the inability to smell a particular substance) becomes more frequent as odorant molecular size increases [18]. Thus, human subjects become increasingly anosmic to large numbers of VOCs as molecular weight increases.

The relationship between aroma quality and odorant molecular properties is harder to quantify in biological systems than with electronic gas sensors due to variability in sensitivities of individuals to specific classes of odorants and individual differences in subjective judgments of how odorants are described or classified [22]. Nevertheless, the measurement of odors from agricultural production areas, industrial facilities, or from municipal solid waste (MSW) landfills is usually a legal requirement for Environmental Protection Agency (EPA) compliance monitoring, planning, site expansion and review of operational practices. Thus, specific methods and practices have been developed for subjective quantification of odors from MSW landfills by regulators, operators and the community for purposes of monitoring, planning and testing [23]. By comparison, individual sensors in the sensor array of e-nose devices can be designed and selected for sensitivity to specific classes of VOCs or VICs based on the chemical nature of odorants such as the types and numbers of chemical functional groups or elements present in odorant molecules. The presence of specific functional groups in analyte gases and the carbon-chain length (molecular weight or size) of aliphatic VOCs from different chemical classes is correlated with odor detection threshold (ODT), but not in rigid-molecule (e.g., cyclic planar and aromatic compounds) [24]. Electronic-nose odor-monitoring systems offer several advantages over human detection. E-nose devices are more sensitive to gas analytes (have much lower ODTs), offer greater potential discrimination of individual gases present (especially when several different analyte-specific e-noses are used simultaneously), and are not subject to operator fatigue as are human monitors.

An important final consideration for designs of e-nose systems for particular agricultural, industrial, and forestry applications is the incidence and frequency of false classifications that occur in association with different gas analyte types and what error rates are acceptable in e-nose discriminations. Random noise in e-nose outputs from the sensor array is one potential source of false classifications. Goodner *et al.* [25] found noise-based false classifications could be minimized by increasing samples sizes, using a minimum number of variables (features) when developing classification models to avoid over-fitting data, making sure the ratio of data points to variables is at least six to prevent over-fitting classification errors, and using different data points (for model validation) other than those used in generating the model. Various algorithms also have been employed to select variables and build predictive data-regression models to improve odorant discriminations and model-validation methods [5,26–28].

False-positive determinations of the presence of specific gas analytes can be as serious as false-negative determinations. The failure to detect toxic gases that may be present in the environment can lead to human fatalities and deaths of farm animals. False-positive indications can result in the implementation of unnecessary pollution control measures or expensive adjustments in industrial processing controls leading to significant economic losses. Thus, selection of the proper sensor array (matched to the specific gas analytes to be detected) and periodic calibration of e-nose monitors is necessary to maintain effective and accurate monitoring of output data from e-nose devices.

## 3. Roles of Electronic-Noses in Modern Agricultural Development

Electronic-nose devices are utilized in a wide range of agricultural industries to perform a multitude of functions ranging from quality-control monitoring of agricultural and forestry products, monitoring industrial-process controls, food production and storage systems, indoor air-quality control, detection of environmental hazards, gaseous and liquid effluents and other factory waste releases. The most common applications of electronic noses in agriculture are to monitor food quality and production processes, detect crop diseases, and identify insect infestations [1]. Some less common uses for e-nose devices include the detection of explosive gases [29], determining the niche-roles of organisms in forested agro-ecosystems [30], monitoring plant physiological processes [31,32], and identifying plants or for plant classifications via chemotaxonomy based on plant volatiles, including essential oils [30].

Plants utilized in agriculture and forestry release VOCs as a byproduct of normal physiological processes. The specific VOCs produced and the quantities released are indicative of both crop and field conditions. Many factors including humidity, available moisture, light, temperature, soil condition, fertilization, insects, and plant diseases may affect the release of VOCs from agricultural plants. Thus, monitoring VOCs released from plants provide indications of plant health, growing conditions, presence of environmental stresses, and the presence of adverse factors that may affect plant growth, production and crop yields.

Product and sample analyses with e-nose devices are accomplished by the detection of headspace volatiles or gaseous VOCs in sampled air, released from organic and inorganic chemical sources associated with the various types of agro-production systems. The following sections provide more specific details of e-nose uses involving specific applications in individual agricultural sectors.

### 3.1. Electronic-Nose Applications within Specific Agricultural Sectors

Electronic-nose devices offer numerous potential applications in agriculture including such diverse uses as the detection of pesticide residue levels on crops or in the environment, industrial applications including detection of gas-leaks and toxic gas emissions, and for homeland security as an early warning system for bioterrorism. Some of the most common applications of e-noses from a wide range of agricultural sectors are listed in Table 3.

Agronomic uses of e-nose devices have included crop-protection applications in the field to detect hazardous chemicals and microbes (e.g., chemical or biological agents of bioterrorism) as well as pesticides on plant foliage [2,33], making selections of plant cultivars of individual crop types for cultivation [34,35], and to monitor plant cell cultures for growth and behavior [36]. Related e-nose applications are found in horticulture involving similar tasks of aseptic plant tissue culturing in the laboratory and cultivation of plant stocks in the greenhouse environment for commercial production of ornamental (e.g., flowers, landscape shrubs) and food (crop) plants.

Electronic-noses have been utilized for several botanical applications involving the detection and monitoring of volatile biogenic gas emissions and floral odors to determine season variations in plant emissions [37,38], for identification of plant host-defense mechanisms, and for plant identifications based on nonfloral volatiles [30]. Dudareva and Pichersky [39] reviewed the potential of metabolic engineering to modulate the volatile profiles of plants to enhance direct and indirect plant chemical defenses and to improve scent and aroma quality of flowers and fruits. Advances in metabolic engineering techniques have provided a better understanding of the biochemical pathways involved in the biosynthesis of volatile secondary metabolite compounds, facilitating the identification of the plant genes and enzymes involved as well as the chemical structures of a large number of new plant volatiles [40–43]. Plants produce a large diversity of low molecular weight VOCs known as secondary or specialized metabolites. At least 1% of these plant secondary metabolites (PSMs) are lipophilic molecules (consisting primarily of terpenoids, phenylpropanoids/benzenoids, fatty acid and amino acid derivatives) with low boiling points and high vapor pressures at ambient temperatures. Plant secondary metabolites are released from all parts of the plant (e.g., roots, stems, leaves, flowers and fruits) into the atmosphere. The primary functions of PSMs are to defend plants against insect herbivores and microbial pathogens, attract pollinators, facilitate seed dispersers, promote the growth of beneficial animals and microorganisms, and serve as chemical signals involved in plant-plant and plant-herbivore interactions. Thus, PSMs are important volatiles that contribute to plant defenses as well as survival and reproductive success in natural ecosystems. Production of PSMs by crop plants also has a significant impact on agronomic and commercial plant characteristics, crop yield and food quality. Consequently, the modification of PSM-volatile production via genetic engineering has the potential to make crop plants less attractive to herbivore enemies and improve the traits of cultivated plant species.

The utilization of metabolic engineering technologies to modify PSM-volatile spectrums of plant presents an enormous potential for plant improvement because of the great contribution of volatile secondary metabolites to plant reproduction, defense and food quality [39]. Electronic-noses offer significant assistance to this effort by providing the capabilities to monitor and identify the sources of PSM-volatile mixtures released from specific plant species [30].

### 3.2. Electronic-Nose and Electronic-Tongue Applications in the Food Industry

The largest proportion of e-nose applications within agriculture over the past twenty-five years has been in the food-production industry. There has been considerable interest in the use of electronic devices for the sensing of food aromas for several major applications in the food industry. Electronic noses are needed as objective, automated sampling systems to monitor food quality and characterize the aromas of multiple food products simulaneously to determine whether the production system is running to specifications—without requiring human sensory panelists, lengthy analytical methods or data interpretations [127]. In an automated food production system, electronic noses serve to rapidly obtain quality-classification information on food products to maintain product quality, uniformity, and consistency based on aroma characteristics. Specific VOCs released from food constituents are responsible for the characteristic aroma of food products. Other uses of e-noses in the food industry include: quality assurance of raw and manufactured products, monitoring of cooking processes, fermentation processes, mixing, flavoring, blending and product-packaging interactions, determining food freshness and aging in storage, evaluating the maturation and ripening of wine, cheese, and meat products. The e-nose assessment of food freshness and spoilage during processing, packaging, and storage are particularly important for assuring that the final products presented for human consumption are of sufficient quality to be salable in commercial markets.

E-noses are used in the flavor and food industries for many of the same tasks employed in the cosmetics and perfume industries. The differential volatilities of chemical species that compose the complex aromas released from commercial food products are given major consideration in product development. The food and beverage industries, like the perfume or scent industries, seek to manage and manipulate product aromas for commercial or market-share advantages. Thus, the continuous search for attractive or pleasing aromas and flavors to enhance food products is a major preoccupation in the food and beverage industries. The characteristics and qualities of complex aromas, composed of a widely diverse mixture of volatile chemical constituents including VOCs that collectively produce the unique olfaction sensation that defines a specific product, are key attributes receiving the greatest attention in product-development research [4].

Potentiometric electronic-tongue (e-tongue) instruments for evaluating and quantifying the quality of taste characteristics of food products are functionally analogous instruments to electronic-noses that focus on the olfactory or aroma characteristics of foods. Some diverse applications of electronic-nose and e-tongue technologies in the food industry are listed in Table 4. E-tongues have been applied to the food and beverage industries in many of the same functions as e-noses, such as for food-taste monitoring, classification, grading, quality assessments, and predictions of human taste-test results for commercial food and beverage products. Hruskar *et al.* [128] utilized a potentiometric e-tongue, consisting of seven sensors and an Ag/AgCl reference electrode, to effectively monitor taste changes in probiotic fermented milk in storage, to classify probiotic fermented milk according to flavor, and to predict sensory characteristics and their relationship to the quality of the fermented milk as measured by human consumers.

They employed various pattern-recognition techniques, including multivariate data processing based on principal component analysis (PCA) for monitoring changes in the four types of fermented milk (plain, strawberry, apple-pear, and forest-fruit) during storage, and partial least squares regression (PLS) with artificial neural networks (ANNs), to estimate and predict human sensory panel evaluation results. Correct classification of the four fermented milk types ranged from 87–95% correct identification with a high level of correlation for ANN (r^2^ = 0.998) and PLS (r^2^ = 0.992). Sensor analysis and food classification using potentiometric e-tongues have been applied to many other similar functions to qualify taste characteristics in the food and beverage industries [49,89,143,147,148,181,183].

The cognitive mechanisms that control human sensory perceptual interactions between olfaction and taste have been thoroughly studied. Olfaction has a strong influence on taste and trigeminal perceptions and modulates perceptual taste/taste and taste/trigeminal sensory interactions, suggesting a multiplicity of overlapping olfactory/trigeminal/taste perceptual interactions to foods with complex flavors [4]. Generally, odor-taste interactions are regarded by the scientific community to be the result of associations experienced and committed to memory following episodes of exposure to foods without any involvement of explicit attention or learning [313–315]. Perceptual interactions between olfaction and taste have been extensively explored in aqueous systems. Initial studies reporting perceptual interactions between olfaction and taste showed that tastes perceived to be attributed to ethyl butyrate and citral odorants by test subjects disappeared when the retronasal olfactory was prohibited by closure of the nasal passages [316,317]. These complex sensory interactions between olfaction and taste have been explored in electronic-sensor research by combining the use of electronic-noses and electronic-tongue technologies to assess the aromas and flavors of specific foods [27,49,138,181,201]. Additional reviews of e-nose and e-tongue applications in the food industry have been published previously [4,318–320].

## 4. Electronic-Nose Applications in Forestry

Tree sap-flow sensors, consisting of cylindrical thermocouples and heater probes for estimating plant transpiration [321], are important instruments for assessing the physiological state of forest trees to determine the presence of drought stresses and to measure wood-moisture content. This information is essential for making forest management decisions such as estimating the proper time for tree harvests. The primary intent of physiological measurements is to monitor physical parameters that are indicators of the health of individual trees. Similarly, electronic-nose devices have been used to determine the presence of damaging insects in wood (e.g., termites) [61], to identify tree diseases [106], and detect other microbial pests that have significant impacts on the present status of forest-stand health and future tree merchantability following tree harvests. Visual assessments to confirm plant-health status, determined with e-nose instruments, also are possible via image analysis of plant symptoms using smart optical sensors [322].

Wilson *et al.* [2] first applied e-nose technologies to plant pathology for the diagnosis of tree diseases, particularly those caused by phytopathogenic microbes, such as vascular wilts [107] and bacterial wetwood, and for the detection and identification of wood decay fungi, causal agents of wood rots in living trees. Subsequent studies have demonstrated the capabilities of several e-nose instruments to detect specific types of wood decays, *i.e.*, those caused by particular wood decay fungi, in different host wood species [86]. The early detection of incipient wood decays in trees with e-noses is particularly important in forested urban environments where tree failures, e.g., breakages of major limbs or the main truck, can cause significant damage to property or result in human fatalities [120,323].

The proper identification of wood types and characteristics has many important applications in forestry, forest management and production, and forest science. Wood type and composition affects the microenvironmental characteristics of forested ecosystems, the types of flora, fauna, and microbes present, the relative utilization of the wood as a food and habitat base, and the quality of forest products manufactured from various wood types present in a forest stand.

Three species of conifers predominate in the forest stands of eastern Canada, including black spruce (*Picea mariana*), balsam fir (*Abies balsamea*) and jack pine (*Pinus banksiana*). The quality of pulp and paper produced from wood chips of these three species is determined by the proportion of wood types present in the wood chip mixture for each batch. Consequently, a determination of the composition of wood types present in the mixture is a prerequisite to obtaining an accurate assessment of expected product (paper) quality. Garneau *et al.* [324] utilized a Cyranose 320 e-nose, containing a sensor array with 32 thin-film carbon black composite (CBC) sensors, to discriminate between the odor signatures (fingerprints) of wood chip mixtures (in each sample batch) based on wood-type composition derived from either sapwood or heartwood. Unknown samples were identified at high levels of confidence using CPA and comparisons against aroma reference databases created from known wood-chip mixtures of different wood-type proportions.

Identifications of wood types based on unique mixtures of wood volatiles also are useful for determining niche-functions of microbes and micro-invertebrates in forested ecosystems and in studies of chemotaxonomy [30,85,126]. Such information facilitates understanding of the operations and interactions between organisms in ecosystem microclimates, facilitating multi-use forest management decisions. Headspace volatiles from woody plant parts provide valuable chemotaxonomic data to indicate relatedness between plant species within and between plant families that often support genetic (DNA sequence-homology) data.

The specificity of e-nose identifications of wood samples is so precise that e-nose aroma signatures may even be used to identify individual logs that are inventoried from a tree harvest [325,326]. Log tracking with e-nose devices has been developed to help counter high-value log theft that has become increasingly common on public lands in the United States, and to facilitate inventory-accounting of harvested logs from the forest stand to the lumber mill. During log-sniffing procedures, e-noses also may be used to improve the efficiency of logging cuts in log-harvesting operations by detecting bole sections with decay or defects and guiding laser scanners of logging harvester machines [327]. Similarly, e-noses may be used in the logging yard and in the lumber-cutting line of commercial saw mills to detect wood decays and defects in logs to increase the efficiency of saw cuts by minimizing lumber-defect losses (cull volume).

There are several important functions that e-nose instruments play within the manufacturing sector of the forest products industry. E-nose applications in forest-products manufacturing include industrial processing controls, particularly for monitoring of chemical and biochemical processes to adjust machinery controls [87–89], quality control [90], and waste management [5]. Federal regulations require personnel at industrial processing plants to monitor, detect, and control hazardous waste emissions, including gas releases of malodorous effluents and air pollutants from industrial facilities, lumber and paper mills that operate within the forest products industry. Electronic noses serve a very significant function in keeping forest-products manufacturing plants safe for the environment and surrounding communities.

## 5. E-Nose Instrument Types Used in Agriculture and Forestry Applications

A wide range of e-nose instrument types are utilized in the agricultural and forestry industries to perform many diverse functions and applications to facilitate the multitude of steps and processes involved in the production of plant-based products (Table 5). The majority of these applications have involved the use of MOS and CP-type sensors, but other e-nose sensor types (CBC, CO_2_, ECS, MOSFRT, QMB, SAW, and SnO_2_ sensors) have been used to detect certain specialized types of gas analytes.

The major application sectors to which e-nose gas detections have been applied within the agricultural and forestry industries are in such key areas as crop and food production, chemotaxonomy, environmental protection and monitoring, manufacturing process controls, plant pathology, quality control and quality assurance (QA/QC), waste management, and wood identifications.

Testing the aroma qualities and characteristics of manufactured plant products resulting from specialized manufacturing processes is among the most important utilities afforded by the use of e-nose devices in agriculture and forestry. E-noses are capable of discriminating very subtle differences in the aroma characteristics of manufactured food and fiber products which affect aromatic favorability qualities (discerned by consumers) that often determine their choices of preferred product brands. For example, many different coffee brands are available in commercial food markets of most developed countries. The aroma constituents of coffee are very complex involving hundreds of VOCs with a wide range of functional groups [342]. Studies of the most significant constituent compounds accounting for the characteristic coffee aroma have indicated that about 29 VOCs were most responsible for the roast and ground coffee aroma of which only 13 had a particularly important contribution to coffee aroma [152,343]. Thus, no single compound was found that could be considered most responsible for the typical flavor of roasted and ground coffee.

Routine analyses frequently are performed on coffee aromatic extracts to evaluate the effectiveness of the extraction methods used in rendering a quality coffee aroma. A good extraction method is expected to provide an extract with sensory characteristics very close to the aroma of ground coffee beans prior to extraction. Sarrazin *et al.* [344] evaluated five different extraction methods on three coffee brands: supercritical-fluid extraction with carbon dioxide, simultaneous distillation extraction, oil recovery under pressure, and vacuum steam-stripping with water (or with organic solvent), to compare the resulting coffee aromas derived from these extraction methods. Arabica Colombia coffee also was used for comparison at three different roasting levels: green coffee, light-roasted and medium roasted. By sensory testing, they found that the vacuum steam-stripping method with water provided the most representative aroma extract for all three coffees.

The specific compounds responsible for the characteristic aromas of many other food products similarly have been determined to identify the target chemicals that should be included in aroma-recognition libraries for e-nose or e-tongue tests to evaluate food processing methods and product brands. Precise chemical analyses of the aromatic compounds most representative and responsible for the characteristic aromas associated with common fruits have been determined for citrus [345], pineapple [346], watermelon [347], and wine (fermented grapes) [348].

Lorenz *et al.* [89] utilized an electronic tongue to determine the taste-masking effectiveness of pharmaceutical formulations compared to placebos. Just like plant-based food products, oral pharmaceutical products that reside in the mouth long enough to be tasted must be palatable. Palatable attributes include appearance, taste, smell, and texture. Palatability affects compliance (patient use of a prescribed drug) and dictates whether a therapeutic outcome is attained. Palatability of the drug product must be given careful consideration to achieve optimal effectiveness because the drug cannot work if the patient does not take the medication. Palatability also affects commercial success of a drug product because drug formulations with higher palatability have a greater chance of being prescribed by physicians when there is a choice between several products with similar efficacy and safety profiles. The electronic tongue used in this study was an Alpha MOS Astree II with 7 sensors consisting of MOS Field Effect Transistors (MOSFET), similar to ion-selective FET, but coated with a proprietary membrane. Specific chemical compounds were embedded in the co-polymer coating to impart cross-selectivity/cross-sensitivity. The sensors were made with a polymer matrix, plasticizer and various sensitive materials (e.g., alcoholic or hydrophobic ionophores). The data were collected using a Ag/AgCl reference electrode.

## 6. E-Nose Uses in Combination with other Sensing Technologies

The potential to utilize electronic-nose devices in concert with other electronic sensing instruments and new analytical detection methods for additive or synergistic benefits are considerable. The following discussion provides some recent examples of feasible applications, showing how other detection methods might be used in cooperation with e-noses to yield better, more detailed information so critical to effective decision-making required in all phases and types of agricultural and forestry production systems.

### 6.1. DNA Microarrays

E-nose devices have been used extensively to detect pathogens present in fish products [4,264–268]. However, other detection technologies such a DNA microarrays are becoming increasingly useful in helping to simultaneously identify the specific microbes or combination of microbes responsible for fish diseases. Chang *et al.* [349] recently combined the use of 16S rDNA PCR and DNA hybridization technology to construct a microarray for the simultaneous detection and discrimination of eight fish pathogens (*Aeromonas hydrophila*, *Edwardsiella tarda*, *Flavobacterium columnare*, *Lactococcus garvieae*, *Photobacterium damselae*, *Pseudomonas anguilliseptica*, *Streptococcus iniae* and *Vibrio anguillarum*) most commonly encountered in fish aquaculture. The microarray consisted of short oligonucleotide probes (30 mer), complementary to the polymorphic regions of 16S rRNA genes of the target pathogens. Target DNA that annealed to the microarray probes were reacted with streptavidin-conjugated alkaline phosphatase and nitro blue tetrazolium/5-bromo-4-chloro-3′-indolylphosphate, *p*-toluidine salt (NBT/BCIP), resulting in blue spots (color reaction) that was easily visualized by the naked eye. Testing performed on 168 bacterial strains showed that each probe in the microarray consistently identified its corresponding target strain with 100% specificity. The microarray detection limit was estimated to be about 1 pg for genomic DNA and 103 CFU/mL for pure pathogen cultures. These results demonstrated the feasibility of using DNA microarrays to facilitate the simultaneous diagnostic testing for multiple fish pathogens. Zhang *et al.* [350] summarized the current status of microarray technology for the detection and analysis of chemical contaminants in foods.

### 6.2. Biosensors

The common use of e-noses to detect microbial toxins produced by human pathogens in foods [307–311] may be improved by the additional detection of the specific microbial strains of human pathogens (such as *Escherichia coli*) known to cause the most damage to humans that consume contaminated foods. Liu *et al.* [351] multiplexed an electrochemical DNA biosensor for the detection of a highly specific single-nucleotide polymorphism (SNP) within the β-glucuronidase gene (uidA), characteristic of the most toxic strain of *E. coli*. A 16-electrode array was applied with an oligonucleotide-incorporated nonfouling surface (ONS) on each electrode for the resistance of unspecific absorption. The fully matched target DNA templated the ligation between the capture probe, assembled on gold electrodes and the tandem signal probe with a biotin moiety, which was transduced to peroxidase-based catalyzed amperometric signals. They demonstrated the potential practical use of the ONS-based electrochemical DNA biosensor using a SNP on the β-glucuronidase gene (uidA) of *E. coli* (T93G) to screen food lots and detect the presence of the most harmful (O157:H7) *E. coli* strain in order to help prevent possible life-threatening *E. coli* outbreaks due to consumption of contaminated food lots.

Label-free optical detection systems for industrial small-molecule chemical screening applications have gained popularity during the past decade within many industries. Microplate-based biosensor systems hold the promise to match the throughput requirements for industrial uses without compromising data quality, thus representing a sought-after complement to traditional fluidic systems. Geschwindner *et al.* [352] reviewed the application of the two most prominent optical biosensor technologies, namely surface plasmon resonance (SPR) and optical waveguide grating (OWG), in small-molecule screening. These methods offer good complimentary support for e-nose sensors to monitor industrial chemicals in manufacturing processes.

Microsensing systems using biotic sensor components, such as optical fiber biosensors, are in high demand because of their lower cost and usefulness as tools for measurement and analysis in the fields of biorobotics, healthcare, pharmaceuticals, environmental monitoring, and military defense as well as in various agricultural applications, such as disease diagnosis, food testing, and environmental detection of biological agents (homeland security). Thus, optical biosensors compliment the same detection objectives of e-nose instruments. Zhang *et al.* [353] recently proposed a new fiber surface-modification methodology using gold nanoparticles to increase the sensitivity of fiber-optic plasmon resonance biosensors.

### 6.3. Chemical Aptasensors

The compatible marriage between conducting polymer (CP) technologies of electronic noses and modified electrodes using nanoparticles, derived from electrochemical (electrode) technologies, has resulted in the development of chemical aptasensors (electrochemical biosensors) consisting of CP nanocomposite materials produced by the electropolymerization of CPs onto specialized nanoparticle electrodes. Nanocomposites containing inorganic nanoparticle and CPs allow current flow with unique electrical and optical properties, compared to CPs or metal nanoparticles alone [354,355]. The electrocatalytic properties of nanoparticles are enhanced by the favorable environment supplied by the CP-polymeric matrix [356]. Conducting polymers exhibit unique properties such as catalysis, conductivity, biocompatibility, and the ability to act as an electrical plug connecting the bio-recognition element to the surface of the electrode [357–359].

One major class of environmental contaminants called Endocrine Disrupting Chemicals (EDCs), named for the disruptions these chemicals cause to normal functions of the endocrine system, has become an important research topic in the field of environmental science because EDCs cause adverse effects on humans and their progeny, as well as on many other organisms in natural environments. EDCs are ubiquitous because of their abundant use in many industrial and agricultural applications [360]. Most EDCs are synthetic organic chemicals introduced into the environment by anthropogenic sources, but they can also be naturally generated by the estrogenic hormones 17β-estradiol and estrone in humans exposured to EDCs especially via drinking water. Consequently, the detection of these chemicals in humans and the environment is necessary to protect public and environmental health. Olowu *et al.* [361] developed a simple and highly sensitive electrochemical DNA aptasensor with high affinity for endocrine-disrupting 17β-estradiol. Poly(3,4-ethylenedioxylthiophene) (PEDOT), doped with gold nanoparticles (AuNPs), was electrochemically synthesized and employed for the immobilization of biotinylated aptamer to detect the 17β-estradiol target. The aptasensor distinguished 17β-estradiol from structurally-similar endocrine disrupting chemicals, demonstrating specificity to 17β-estradiol. The detectable concentration range of the 17β-estradiol was 0.1 nM–100 nM, with a detection limit of 0.02 nM.

### 6.4. Electronic Tongues

Electronic noses have been used in combination with electronic-tongues for many applications primarily in the food industry [27,49,138,181,201]. However, potentiometric e-tongues have been employed in a wide range of other applications in agriculture and forestry. Some examples of e-tongue applications include the detection and analysis of alkaline ions [362–364], anions [365], ascorbic acid [366], environmental pollutants monitoring [367], heavy metal ions [368], nitrates [369], oxidizable compounds [370], paper mill effluents [371], pesticides [372], and phenolic compounds [373] in liquids or industrial-processing solutions. Gutierrez *et al.* [374] used an e-tongue to monitor fertigation (*i.e.*, application of fertilizers in irrigation water) nutrients applied for greenhouse cultivation (plant propagation). In agricultural food analyses, e-tongue sensors often utilize a lipid membrane as a taste element to measure electrical charge potential across the membrane when analytes (taste) molecules come in contact with it. The detection limit of the e-tongue sensor may be optimized by adjusting the concentration of the lipid in the membrane [186].

Vlasov *et al.* [375] provided an early review of the developmental history of potentiometric sensors as an analytical tool, over the past century, describing advances from single-ion sensors to new multisensor arrays for liquid (solution) analysis that utilize advanced mathematical procedures for signal processing based on pattern recognition (PARC) and multivariate analysis including ANNs and PCA. More recent reviews provide further details of e-tongue developments [376–378].

### 6.5. Electroconductive Hydrogels

Electroconductive hydrogels are composite biomaterials made of polymeric blends combining conductive electroactive polymers (CEPs) with highly hydrated hydrogels. They bring together the redox-switching and electrical properties of CEPs with the small-molecule transport and compatibility of cross-linked hydrogels [379]. CEPs often are incorporated into biosensors to detect chemical species such as proteinaceous antigens, metabolites, enzyme substrates, and ssDNA fragments [90]. The capability of detecting proteins, enzymes, and DNA fragments is most useful for sensing the presence of toxins and microbial contaminants in foods, beverages, and drinking water. Park *et al.* [380] recently developed a suspension protein microarray using shape-coded polyethylene glycol (PEG) hydrogel microparticles for potential applications in multiplex and high-throughput immunoassays. Two different mixtures of hydrogel microparticles with different shapes, immobilizing IgG (circle) and IgM (square), were prepared allowing simultaneous detection of two different target proteins without cross-talk using the same fluorescence indicator because each immunoassay was easily identified by the shapes of hydrogel microparticles.

Many other examples show the potential for using e-nose instruments in combination with other electronic-sensing devices to help confirm gas-detection determinations for specific application areas [90]. Spinelli *et al.* [381] evaluated the use of a near infrared (NIR) instrument in combination with an electronic nose system for the early detection of fire blight (disease) in pears. The e-nose system detected the disease prior to symptom development by the distinctive olfactory signature of volatiles released as early as six days after infection. Sankaran *et al.* [382] reviewed other advanced techniques and instruments for detecting plant diseases which might be used in combination with electronic noses for disease diagnoses.

## 7. Conclusions

Electronic-nose devices have been utilized in a wide diversity of applications in the agriculture and forestry industries to improve the effectiveness, efficiency and safety of processes involved in the production of quality food and fiber plant-based products while at the same time helping to avoid the adverse effects of chemical byproducts on human health and the release of toxic chemical gases and effluents into the environment. The challenges for the future are to further develop e-nose technologies to expand on potential applications in these natural plant-production sectors by exploring several new key areas of scientific R&D including the development of smaller, portable devices more applicable to field use, simpler application-specific instruments at lower costs, new sensor types and algorithms for more effective gas-detection and discriminations, and the discovery of new, problem-solving applications requiring gas-sensing tasks within plant-product industries. There is also a large potential for the integration of e-nose uses with other electronic-sensing instruments for cooperative and synergistic applications, providing more useful information for decision-making by resource, industrial and plant-production managers. This work will require the development of new specific e-nose technologies with expanded sensor capabilities and thorough efficacy testing in real, end-user settings.

Recent advancements in e-nose designs and methods could lead to improved gas-analyte detection. For example, Brudzewski *et al.* [383] reported on an improved e-nose that combines two identical or very similar sensor arrays. Analyte aromas were analyzed independently by the sensor arrays and the difference between sensor output signals from the arrays was subject to 2-dimensional convolution, greatly enhancing the sensitivity of the e-nose. Choi *et al.* [384] developed new data-refinement and channel-selection methods for vapor classification to reduce background noise in the data and distinguish the portion of the data most useful for discriminations with a portable e-nose system. Data refinement improved data clustering of different aroma classes and classification performance. They also designed a new sensor array that consisted only of the useful (most aroma-discriminative) channels. They analyzed data channels from individual sensors by evaluating discriminative power using the mask feature in data refinement. By this process, the new sensor array had improved classification rates and efficiency in data computation and storage.

Finding new ways to improve e-nose performance through the use of better or more target-specific sensors and sensor arrays, pattern-recognition algorithms, data analysis methods, and sensor architecture and micromorphology should significantly widen the range of gas-sensing capabilities and applications of e-noses in agricultural and forestry plant-product industries. Several studies have shown how nanostructures may be applied to e-nose sensors to improve instrument performance. Twomey *et al.* [385] devised techniques using a combination of microfabrication techniques, e-beam evaporation and pulsed-laser deposition, to apply coatings on an electronic-tongue device that contained all of the electrodes integrated on a silicon die to improve robustness and reproducibility of the device. Sun *et al.* [386] recently reviewed some of the ways that sensitivity, selectivity, response speed, and performance of MOS sensors could be improved such as through changes in the morphology and structure of sensing materials, including modifications in particle size, shape, porosity and metal-doping. When the particle size of metal-oxide sensor coatings is close to or less than double the thickness of the space-charge layer, the sensitivity of the sensor will increase remarkably (known as the “small-size effect”), yet the small size of metal oxide nanoparticles will be compactly sintered together during the film-coating process, a significant disadvantage for analyte gas diffusion. Metal doping is particularly useful in enhancing catalytic activity and modulating the intrinsic electrical resistance of the metal-oxide sensor coating. Zhang *et al.* [76] found that unmodified multi-walled carbon nanotubes (MWNTs) and those modified by atmospheric pressure dielectric barrier discharge (DBD) air plasma improved gas sensor sensitivity, response time, and selectivity for H_2_S, but not for SO_2_ detection. Chen *et al.* [387] reviewed the recent development of e-nose systems based on metal oxide nanowires with great potential for the improvement of sensor selectivity. They also discussed the use of 1-D metal oxide nanostructures with unique geometric and physical properties for chemical-sensing applications. Chemical sensors composed of a wide range of pristine 1-D metal oxide nanostructures, such as In_2_O_3_, SnO_2_, ZnO, TiO_2_, and CuO, have exhibited good sensitivity for the detection of important industrial gases.

Electronic noses with diverse sensor arrays are responsive to a wide variety of possible gas analytes and have a number of advantages over traditional analytical instruments. Electronic nose sensors do not require chemical reagents, have good sensitivity and specificity, provide rapid repeatable (precise) results, and allow non-destructive sampling of gas odorants or analytes [388]. Furthermore, e-noses generally are far less expensive than analytical systems, easier and cheaper to operate, and have greater potential for portability and field use compared with complex analytical laboratory instruments [90]. Thus, electronic noses have far greater potential to be customized for unskilled laborers and for innumerable practical and mechanized applications in the agricultural and forest-products industries. However, some disadvantages of e-nose sensing include problems with reproducibility, recovery, negative effects of humidity and temperature on sensor responses, and inability to identify individual chemical species within gas samples. Thus, electronic noses probably will never completely replace complex analytical instruments, but offer quick real-time detection and discrimination solutions for applications requiring accurate, rapid and repeated determinations [90]. Such applications are increasingly common and required for highly-mechanized industrial manufacturing processes. The real time, rapid-analysis capabilities of new portable e-noses are not only required but expected operating capabilities to accommodate the fast-paced activities and mechanized processes of modern industries.

New sensing technologies emerging from R&D are beginning to yield new ways of improving on e-noses and EAD capabilities through interfaces and combinations with classical analytical systems for rapid identification of individual chemical species within aroma mixtures. E-nose instruments are being developed that combine EAD sensors in tandem with analytical detectors such as with fast gas chromatography (FGC) [389]. More complicated technologies such as optical gas sensor systems may improve on traditional e-nose sensor arrays by providing analytical data of mixture constituents [390]. Similar capabilities for identifying multiple components in liquid mixtures are now possible using electronic tongues.

Very recent literature on e-nose applications in agriculture and forestry provide some indications of future trends in R&D and industrial uses within these areas. The strongest trend appears to be the expanded utilization of e-nose devices as a monitoring tool in the food industry, assuring the safety and quality of consumable plant products, continuing with the development of new methods to detect chemical contaminants [350,391], adulterations with baser elements [190,259,260], food-borne microbes and pathogens [263,351,392–395], and toxins [84,311,396] in crops and food products. Similarly, new food-analysis e-nose methods are being developed to detect changes in VOCs released from foods and beverages in storage to assess shelf-life [346,397,398] and quality [185,206,399–403], and for chemical analyses [404,405], classifications [227,232,346,406,407], and discriminations [162,218,228,408] of food types, varieties and brands. Electronic-nose applications to detect plant pests in preharvest and postharvest crops and tree species continue to expand to include new insect [54–61] and disease [111,112,339,409–413] pests, primarily microbial plant pathogens, beyond those originally reported by Wilson *et al.* [2,106,107]. In the macroenvironments adjacent to industrial plants and indoor working spaces within associated food- and fiber-production facilities, e-noses increasingly are being utilized to monitor air quality to detect hazardous chemicals [68–70,76,77,80,414–419], explosives and flammable gases [29,64], pollutants [420–422] and other VOCs that threaten human health. Likewise, malodorous gases produced from point sources, such as agricultural feedlots and paper-production facilities (pulp mills), increasingly are being monitored by e-nose devices to assure that release of gaseous odors and effluents are maintained below offensive and hazardous threshold levels [423–427]. Pesticide residues on food crops, particularly on fresh fruits and vegetables, likely will be monitored electronically with e-noses in the future by Food and Drug Administration (FDA) officials for certification (clearing foods for safe consumption) prior to marketing in groceries and fresh-food stores [33]. Electronic-nose detection of human pathogens on fresh food surfaces also should be possible with the development of portable e-noses having rapid sensor-array detection, analysis and recovery times. E-nose applications involving the identification of agricultural plants [37,126] and animal [306,428] species will become useful for many types of checks for quality and identity controls, verification assurance, health tests, and government-regulation enforcement. Tests of soil health and microbiological activity will provide means of assuring that crop plants are grown in healthful growth environments and in soils free of harmful chemicals or microbes [429]. Finally, electronic-noses are having greater utility in indoor agricultural production within greenhouses, such as for environmental controls of air quality (pollutants) [104], relative humidity [102], fertigation metering [374], and irrigation water quality [80] to assure that ornamental and food crops remain free of biotic and abiotic diseases [110,430].

The potential for future developments and new applications of electronic-nose devices for the agriculture and forestry industries are enormous as new technological discoveries in electronic-sensor design allow for the development of new gas-sensing capabilities for electronic noses. The current trend of developing electronic noses for specific narrower applications will likely continue because such instruments are cheaper and provide greater utility, efficiency, and effectiveness in gas-sensing operations in specialized industrial applications. The efficiency of specialized e-noses is derived from the ability to minimize the number of sensors needed for discriminations by targeting the detection of specific gases which reduces instrument costs, allowing for greater portability through miniaturization. New potential discoveries in sensor materials and technologies will help to expand e-nose capabilities as new products, machines, and industrial processes are developed. These discoveries will lead to the recognition of new ways to exploit the electronic nose to solve many gas-detection problems arising in the agricultural and forestry industries.

## Figures and Tables

**Table 1.** Major types of VOCs in gas mixtures detected with e-noses in agriculture and forestry.

**Volatile chemical types**	**Example compound**	**Chemical structure**	**Common source/use**
Biochemical	pyruvic acid	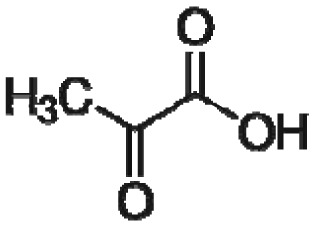	Cellular metabolite
Food products	citrinin	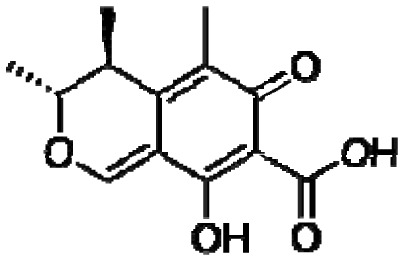	Mycotoxin contaminant
Floral	methyl propionate	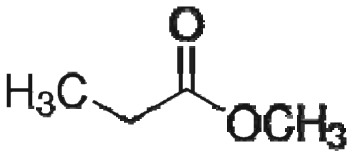	Flower fragrance
Fruit	2-phenylethanol	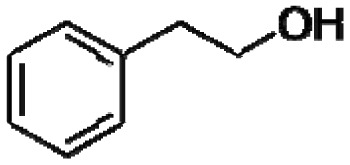	Wine volatile
Microbial	acetic acid	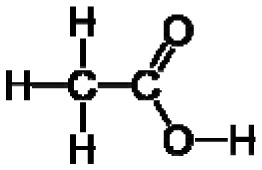	Fermentation product
Pesticides	glyphosate	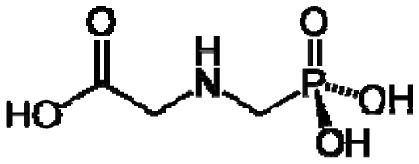	Herbicide
Plant hormones	ethylene	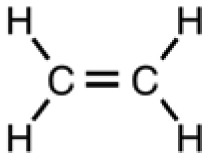	Fruit-ripening hormone
Secondary metabolites	caffeine	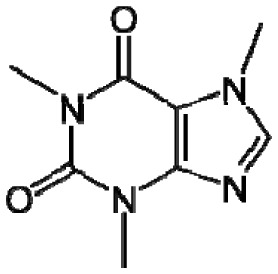	Plant alkaloid
Vegetative	hexenyl acetate	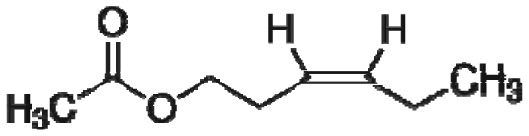	Leaf volatile
Waste	dimethyl disulfide	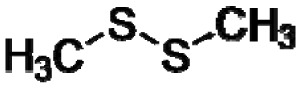	Paper byproduct
Wood	α-pinene	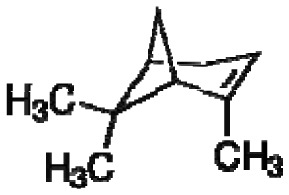	Wood volatile

**Table 2.** Offensive agricultural byproducts with threshold levels for human detection and recognition.

**Chemical odorant**	**Formula**	**Characteristic odor**	**Detection** †	**Recognition** †
Acetaldehyde	CH_3_CHO	Pungent, fruity		2.1 × 10^−1^
Allyl mercaptan	CH_2_CHCH_2_SH	Strong garlic, coffee	1.6 × 10^−2^	
Ammonia	NH_3_	Sharp, pungent		4.7 × 10^1^
Amyl mercaptan	CH_3_(CH_2_)_4_SH	Putrid		
Benzyl mercaptan	C_6_H_5_CH_2_SH	Strong		
Butylamine	C_2_H_5_(CH_2_)_2_NH_2_	Ammonia-like, sour		2.4 × 10^−1^
Cadaverine	H_2_N(CH_2_)_5_NH_2_	Putrid, decaying flesh		
Chlorophenol	ClC_6_H_5_O	Phenolic, medical		
Crotyl mercaptan	CH_3_CH=CHCH_2_SH	Skunk-like	7.7 × 10^−3^	
Dibutylamine	(C_4_H_9_)_2_NH	Fishy		
Disopropylamine	(C_3_H_7_)_2_NH	Fishy		8.5 × 10^−2^
Dimethyamine	(CH_3_)_2_NH	Putrid, fishy		4.7 × 10^−2^
Dimethylsulfide	(CH_3_)_2_S	Decayed vegetables		1.0 × 10^−3^
Diphenylsulfide	(C_6_H_5_)_2_S	Unpleasant		2.1 × 10^−3^
Ethylamine	C_2_H_5_NH_2_	Ammonia-like		8.3 × 10^−1^
Ethyl mercaptan	C_2_H_5_SH	Decayed cabbage	2.6 × 10^−3^	1.0 × 10^−3^
Hydrogen sulfide	H_2_S	Rotten eggs		4.7 × 10^−3^
Indole	C_2_H_6_NH	Nauseating, fecal		
Methylamine	CH_3_NH_2_	Putrid, fishy		2.1 × 10^−2^
Methyl mercaptan	CH_3_SH	Decayed cabbage		2.1 × 10^−3^
Propyl mercaptan	CH_3_(CH_2_)_2_SH	Unpleasant	2.4 × 10^−2^	
Putrescine	NH_2_(CH_2_)_4_NH_2_	Putrid, nauseating		
Pyridine	C_6_H_5_N	Disagreeable, irritating		
Skatole	C_9_H_9_N	Nauseating, fecal	2.2 × 10^−1^	4.7 × 10^−1^
Sulfur dioxide	SO_2_	Pungent, irritating		
Tert-butyl mercaptan	(CH_3_)_3_CSH	Unpleasant, skunk		
Thiocresol	CH_3_C_6_H_4_SH	Rancid, skunk	1.4 × 10^−2^	
Thiophenol	C_6_H_5_SH	Putrid, garlic-like	1.4 × 10^−2^	2.8 × 10^−1^
Triethylamine	C_2_H_5_OH	Ammonia-like, fishy		

† Human thresholds for detection and recognition of odorant gases are measured in parts per million (ppm) in dry air at standard temperature and pressure (STP).

**Table 3.** Major categories of electronic-nose applications within various agricultural sectors.

**Agricultural sector**	**Specific application areas**	**References**
Agronomy/Horticulture	Crop protection	[2,33]
	Cultivar selection & discrimination	[34,35,44]
	Pesticide detection	[33,45–48]
	Plant cell culture	[36]
Biotechnology processes	Monitoring	[49,50]
Botany	Floral odors	[37]
	Plant identification	[30]
	Plant volatiles detection	[30,38,39]
	Taxonomic determinations	[30]
Cell culture	Plant growth	[36]
Chemistry	Chemical detection & identification	[51]
	Classification	[52,53]
Ecology	Niche roles in ecosystem	[30]
	Plant and animal species identification	[30]
Entomology	Detect insects or induced plant volatiles	[54–56]
	Insect identification and plant damage	[57–61]
Environmental hazards	Ecosystem management	[30]
	Explosive vapors	[62–64]
	Health hazards monitoring	[5,65–70]
	Toxic gas detection	[71–77]
	Water contamination detection	[78–81]
Food production	Chemical contaminants	[82]
	Microbial pathogens or toxins	[83,84]
Forestry/Silviculture	Classify/identify wood types	[30,85]
	Forest health protection	[2,86]
	Forest management	[30]
Industrial Processes	Process monitoring control	[87,88]
	Formulation development	[89]
	Quality control	[90]
Microbiology	Discrimination of strains	[91–95]
	Identification of microbes	[96]
	Microbial growth phases	[97]
	Pathogen detection	[98]
	Toxin production	[99]
Monitoring	Enzyme and protein activity	[100]
	Humidity	[101,102]
	Immunoglobulin levels	[103]
	Oxygen levels	[104]
	Plant volatiles	[39]
Physiological conditions	Disease effects on plant physiology	[31,32]
	Fruits	[105]
Plant Pathology	Crop protection against bioterrorism	[2]
	Disease detection and monitoring	[2,106–112]
	Host identification	[30,85]
	Host physiology (pathogenesis effects)	[31,32,105]
	Host resistance	[113]
	Pathogen identification	[2,106]
	Post-harvest decay or rot detection	[114–118]
	Wood decay fungi	[2,86,96,119,120]
	Wood decay types	[2,86]
Waste management	Monitoring malodorous emissions	[23,121–125]
Wood science	Wood identifications	[30,85,126]

**Table 4.** Diverse applications of electronic-nose and e-tongue technologies in the food industry.

**Food industry sector**	**Specific application areas**	**References**
Aroma analysis	Acidity	[129]
	Antioxidants	[130–133]
	Astringency or bitterness	[134–138]
	Beer	[139–143]
	Bioethanol	[144]
	Chemical content analysis	[145–149]
	Coffee	[78,150–153]
	Flavor analysis (taste)	[152,154–162]
	Fragrance or odor analysis	[127,159,163–165]
	Fruit ripening or maturity	[35,116,166–172]
	Fruit and floral volatiles	[37,173,174]
	Fungal volatiles	[175,176]
	General food analysis	[177–183]
	Juice levels in beverages	[184,185]
	Lipid, oils, or fat content	[186]
	Meat	[187,188]
	Milk	[189,190]
	Plant or vegetable oils	[191,192]
	Soft drinks (beverages)	[185,193]
	Soybean	[194]
	Spice mixture composition	[195]
	Storage-condition effects	[196,197]
	Taste analysis and consumer-choice tests	[159,160,198–201]
	Tea	[145]
	Wine	[202–206]
Aroma classifications/discrimination	Alcohol and liqueur	[207,208]
	Apricots	[209,210]
	Baking breads	[211]
	Bitterness of foods & beverages	[134,138,139,212–215]
	Carrots	[216]
	Cheeses	[217,218]
	Chickpeas	[219]
	Citrus juices	[220,221]
	Coffees	[222–224]
	Edible oils	[225–228]
	Floral	[37]
	Food products	[229]
	Grains	[230]
	Herbs	[34]
	Honeys	[231,232]
	Liquids	[139]
	Milk	[161,233]
	Mineral water	[234,235]
	Peaches	[35,82]
	Pears	[236]
	Rice	[237]
	Seeds	[238]
	Soybeans	[44]
	Teas	[145,239,240]
	Tomatoes	[173]
	Volatile organic compounds (VOCs)	[13,50–53,241]
	Wines	[242–246]
Detection & identification	Artificial and natural sweeteners	[247]
Food processing	Control of processing parameters	[87,88]
	Aging of food products	[248–252]
Geographical origin	Cheeses	[217]
	Honeys	[253]
	Olive oils	[254]
	Wines	[255]
	Teas	[256]
Quality control	Adulteration with cheaper components	[192,257–260]
	Contamination with microbes/pathogens	[95,141,230,261–263]
	Coffee	[224]
	Fish	[264–268]
	Foods	[269,270]
	Food storage methods	[271]
	Fruits	[105,272]
Quality control	Fruit maturity	[116,171]
	Fruit decays or rot detection	[114–116,273]
	Meats	[274,275]
	Milk	[276]
	Oxidation	[191,277]
	Off-flavor and off-odor detection	[278,279]
	Product grading and defect detection	[16,279]
	Quality assessments and sorting	[114,115,196,280,281]
	Shelf life before spoilage	[128,282–293]
	Storage age or food freshness	[13,147,174,290,294–306]
	Toxins present in spoiled foods	[99,302,307–311]
	Vegetable flavor	[154,312]
	Wine	[118]

**Table 5.** Electronic-noses used for specific agricultural and forestry applications.

**Applications**	**Electronic-nose**	**Sensors/types** †	**Chemicals detected or uses**	**References**
Crop production	Moses II	8 MOS, 8QMB	Pesticide residues	[328]
	Aromascan A32S	32 CP	Pesticide residues	[33,46]
Environment	BH-114	14 CP	As, Cd, Pb, Zn (in water)	[329]
	Kamina	38 MOS	NH_3_, chloroform	[330]
	ProSAT	8 CP	Diesel oils	[331]
	Cyranose 320	32 CBC	H_2_S, SO_2_, VOCs	[332]
	FreshSense	4 ECS	CO, H_2_S, NH_3_, SO_2_	[266]
Food	EOS 835	6 MOS	Mycotoxin contaminants, fruit variety classifications	[209,333]
	EOS 507	6 MOS	Oxidative status and classify olive oils	[191]
	PEN 2	10 MOS	Mycotoxin contaminants, fish shelf-life and freshness	[305,334,335]
Food	FOX 4000	18 MOS	Alcoholic-beverage off-flavor detection and discrimination	[205]
	Experimental	8 QMB	Water loss in postharvest fruits	[118]
	E-nose	8 MOS	Classify fruit odors by source	[336]
Manufacturing control	Figaro TGS 2600	4 MOS	Continuous monitoring-control of industrial processes	[50]
	Multi-analyzer	10 MOSFET, 19 MOS, 18 SnO_2_, CO_2_	Batch microbial fermentation processes	[337,338]
Plant pathology	Aromascan A32S	32 CP	Disease detection, pathogen ID, wood decay fungi ID	[2,86,106,107]
	LibraNose 2.1	8 QMB	Wood decay and fungi ID	[86]
	PEN 3	10 MOS	Wood decay and fungi ID	[86]
	Cyranose 320	32 CBC	Post-harvest disease detection	[117]
			Wood decay (basal stem rot)	[339]
Plant taxonomy	Aromascan A32S	32 CP	Plant identifications, chemo-taxonomy (classifications)	[30,85,126]
Quality control/quality assurance	A-nose	8 MOS	Detection and classification of coffee sample/batch defects	[224]
	Z-nose 7100	1 SAW	Detecting adulteration in virgin coconut oil	[259]
Waste	EOS 3, 9	6 MOS	Composting gas effluents, alcohols, sulfur compounds	[340]
	PEN 2	10 MOS	Waste-treatment monitoring	[341]
	Aromascan A32S	32 CP	Monitoring odor abatement using a biofiltering system	[125]
Wood	Aromascan A32S	32 CP	Wood identifications, bacterial wetwood detection	[2,30,85,126]

† Number of sensors and sensor type abbreviations: Carbon black composite (CBC), Carbon dioxide sensor (CO_2_), Conducting polymer (CP), electrochemical (EC), Metal oxide semiconductor (MOS), Metal oxide semiconductor field effect transistor (MOSFET), Quartz crystal microbalance (QMB), surface acoustic wave (SAW), and Tin dioxide (SnO_2_), a type of MOS sensor.

## References

[1] Li S., Simonian A., Chin B.A. (2010). Sensors for agriculture and the food industry. Electrochem. Soc. Interfac..

[2] Wilson A.D., Lester D.G., Oberle C.S. (2004). Development of conductive polymer analysis for the rapid detection and identification of phytopathogenic microbes. Phytopathology.

[3] Cagni D., Ghizzoni C., Lanzotti V., Taglialatela-Scafati O. (2000). The role of non-volatile compounds in flavor science: Applications of HPLC-mass spectrometry technique. Flavour and Fragrance Chemistry.

[4] Wilson A.D., Baietto M. (2009). Applications and advances in electronic-nose technologies. Sensors.

[5] Wilson A.D. (2012). Review of electronic-nose technologies and algorithms to detect hazardous chemicals in the environment. Proc. Technol..

[6] Goldstein A.H., Galbally I.E. (2007). Known and unexplored organic constituents in the earth's atmosphere. Environ. Sci. Technol..

[7] Zhu B.L., Xie C.S., Wu J., Zeng D.W., Wang A.H., Zhao X.Z. (2006). Influence of Sb, In and Bi dopants on the response of ZnO thick films to VOCs. Mater. Chem. Phys..

[8] Ge C.Q., Xie C.S., Cai S.Z. (2007). Preparation and gas-sensing properties of Ce-doped ZnO thin film sensors by dip-coating. Mater. Sci. Eng. B.

[9] Itoh T., Wang J., Matsubara I., Shin W., Izu N., Nishibori M., Murayama N. (2008). VOCs sensing properties of layered organic-inorganic hybrid thin films: MoO_3_ with various interlayer organic components. Mater. Lett..

[10] Fernandez C.D., Manera M.G., Pellegrini G., Bersani M., Mattei G., Rella R., Vasanelli L., Mazzoldi P. (2008). Surface plasmon resonance optical gas sensing of nanostructured ZnO films. Sens. Actuator B Chem..

[11] Kim Y.S. (2009). Thermal treatment effects on the material and gas-sensing properties of room-temperature tungsten oxide nanorod sensors. Sens. Actuator B Chem..

[12] Xu J., Jia X., Lou X., Xi G., Han J., Gao Q. (2007). Selective detection of HCHO gas using mixed oxides of ZnO/ZnSnO_3_. Sens. Actuator B Chem..

[13] Zeng W., Liu T.-M. (2010). Gas-sensing properties of SnO2-TiO2-based sensor for volatile organic compound gas and its sensing mechanism. Phys. B Condens. Matt..

[14] Phaisangittisagul E., Nagle H.T., Areekul V. (2010). Intelligent method for sensor subset selection for machine olfaction. Sens. Actuator B Chem..

[15] Che Harun F.K., Covington J.A., Gardner J.W. (2009). Portable e-mucosa system: Mimicking the biological olfactory. Proc. Chem..

[16] Breer H., Kress-Rogers E. (1997). Sense of smell: Signal recognition and transductions in olfactory receptor neurons. Handbook of Biosensors and Electronic Noses: Medicine, Food and Environment.

[17] Peterlin Z., Li Y., Sun G., Shah R., Firestein S., Ryan K. (2008). The importance of odorant conformation to the binding and activation of a representative olfactory receptor. Chem. Biol..

[18] Turin L., Yoshii F., Richard L.D. (2003). Structure-odor relations: A modern perspective. Handbook of Olfaction and Gustation.

[19] Mori K., Shepherd G.M. (1994). Emerging principles of molecular signal processing by mitral/tufted cells in the olfactory bulb. Semin. Cell Biol..

[20] Myth Debunking 1: What Are Aldehydes, How Do Aldehydes Smell and Chanel No.5. http://perfumeshrine.blogspot.com/2008/12/myth-debunking-1-what-are-aldehydes-how.html/.

[21] Rossiter K.J. (1996). Structure-odor relationships. Chem. Rev..

[22] Wise P.M., Olsson M.J., Cain W.S. (2000). Quantification of odor quality. Chem. Sens..

[23] McGinley C.M., McGinley M.A. Odor quantification methods and practices at MSW landfills.

[24] Zarzo M. (2012). Effect of functional group and carbon chain length on the odor detection threshold of aliphatic compounds. Sensors.

[25] Goodner K.L., Dreher J.G., Rouseff R.L. (2001). The dangers of creating false classifications due to noise in electronic nose and similar multivariate analyses. Sens. Actuator B Chem..

[26] Lu Y., Bian L., Yang P. (2000). Quantitative artificial neural network for electronic noses. Anal. Chim. Acta.

[27] Buratti S., Ballabio D., Benedetti S., Cosio M.S. (2007). Prediction of Italian red wine sensorial descriptors from electronic nose, electronic tongue and spectrophotometric measurements by means of genetic algorithm regression models. Food Chem..

[28] Wang X., Ye M., Duanmu C.J. (2009). Classification of data from electronic nose using relevance vector machines. Sens. Actuator B Chem..

[29] Brudzewskia K., Osowskia S., Pawlowskia W. (2012). Metal oxide sensor arrays for detection of explosives at sub-parts-per million concentration levels by the differential electronic nose. Sens. Actuator B Chem..

[30] Wilson A.D., Lester D.G., Oberle C.S. (2005). Application of conductive polymer analysis for wood and woody plant identifications. For. Ecol. Manage..

[31] Anderson L.J., Harley P.C., Monson R.K. (2000). Reduction of isoprene emissions from live oak with oak wilt. Tree Physiol..

[32] Faldt J., Solheim H., Langstrom B., Borg-Karlson A.K. (2006). Influence of fungal infection and wounding on contents and enantiomeric compositions of monoterpenes in phloem of *Pinus sylvestris*. J. Chem. Ecol..

[33] Wilson A.D. (2012). Development of an electronic-nose technology for the rapid detection of agricultural pesticide residues. Phytopathology.

[34] Bernáth J., Novák I., Szabó K., Seregély Z. (2005). Evaluation of selected oregano (*Origanum vulgare* L. subsp. *hirtum* Ietswaart) lines with traditional methods and sensory analysis. J. Herbs Spices Med. Plants.

[35] Benedetti S., Buratti S., Spinardi A., Mannino S., Mignani I. (2008). Electronic nose as a nondestructive tool to characterize peach cultivars and to monitor their ripening stage during shelflife. Postharvest Biol. Technol..

[36] Komaraiah P., Navratil M., Carlsson M., Jeffers P., Brodelius M., Brodelius P.E., Kieran P.M., Mandenius C.F. (2004). Growth behavior in plant cell cultures based on emissions detected by a multisensor array. Biotechnol. Prog..

[37] Fujioka K., Shirasu M., Manome Y., Ito N., Kakishima S., Minami T., Tominaga T., Shimozono F., Iwamoto T., Ikeda K. (2012). Objective display and discrimination of floral odors from *Amorphophallus titanium*, bloomed on different dates and at different locations, using an electronic nose. Sensors.

[38] Villanueva-Fierro I., Popp C.J., Marin R.S. (2004). Biogenic emissions and ambient concentration of hydrocarbons, carbonyl compounds and organic acids from ponderosa pine and cottonwood trees at rural and forested sites in central New Mexico. Atmos. Environ..

[39] Dudareva N., Pichersky E. (2008). Metabolic engineering of plant volatiles. Curr. Opin. Biotechnol..

[40] Degenhardt J., Gershenzon J., Baldwin I.T., Kessler A. (2003). Attracting friends to feast on foes: Engineering terpene emission to make crop plants more attractive to herbivore enemies. Curr. Opin. Biotechnol..

[41] Aharoni A., Jongsma M.A., Bouwmeester H.J. (2005). Volatile science? Metabolic engineering of terpenoids in plants. Trends Plant Sci..

[42] Dudareva N., Pichersky E. (2006). Metabolie engineering of floral scent of ornamentals. J. Crop Improv..

[43] Lücker J., Verhoeven H.A., van der Plas L.H.W., Bouwmeester H.J., Dudareva N., Pichersky E. (2006). Molecular engineering of floral scent. Biology of Floral Scent.

[44] Gregory C., Silva J.B., Wiziacki N.K.L., Paterno L.G., Paniazzi M.C.C., Fonseca F.J. Application of electronic tongue in identification of soybean.

[45] Déjous C., Rebiére D., Pistré J., Tiret C., Planade R. (1995). A surface acoustic wave gas sensor: Detection of organophosphorus compounds. Sens. Actuator B Chem..

[46] Wilson A.D., Oberle C.S. (2004). Identification and discrimination of pesticide residues using electronic aroma detection. Phytopathology.

[47] Obare S.O., de C., Guo W., Haywood T.L., Samuels T.A., Adams C.P., Masika N.O., Murray D.H., Anderson G.A., Campbell K. (2010). Fluorescent chemosensors for toxic organophosphorus pesticides: A review. Sensors.

[48] Mishra R.K., Deshpande K., Bhand S. (2010). A high-throughput enzyme assay for organophosphate residues in milk. Sensors.

[49] Rudnitskaya A., Legin A. (2008). Sensor systems, electronic tongues and electronic noses, for the monitoring of biotechnological processes. J. Ind. Microbiol. Biotechnol..

[50] Trincavelli M., Coradeschi S., Loutfi A. (2009). Odour classification system for continuous monitoring applications. Sens. Actuator B Chem..

[51] Gao T., Tillman E.S., Lewis N.S. (2005). Detection and classification of volatile organic amines and carboxylic acids using arrays of carbon black-dendrimer composite vapor detectors. Chem. Mater..

[52] Tillman E.S., Koscho M.E., Grubbs R.H., Lewis N.S. (2003). Enhanced sensitivity to and classification of volatile carboxylic acids using arrays of linear poly(ethylenimine)-carbon black composite vapor detectors. Anal. Chem..

[53] Tillman E.S., Lewis N.S. (2003). Mechanism of enhanced sensitivity of linear poly(ethylenimine)-carbon black composite detectors to carboxylic acid vapors. Sens. Actuator B Chem..

[54] Henderson W.G., Khalilian A., Han Y.J., Greene J.K., Degenhardt D.C. (2010). Detecting stink bugs/damage in cotton utilizing a portable electronic nose. Comput. Electron. Agric..

[55] Miresmailli S., Gries R., Gries G., Zamar R.Z., Isman M.B. (2010). Herbivore-induced plant volatiles allow detection of *Trichoplusia ni* (Lepidoptera: Noctuidae) infestation on greenhouse tomato plants. Pest Manage. Sci..

[56] Degenhardt D.C., Greene J.K., Khalilian A. (2012). Temporal dynamics and electronic nose detection of stink bug-induced volatile emissions from cotton bolls. Psyche.

[57] Rains G.C., Tomberlin J.K., D'Alessandro M., Lewis W.J. (2004). Limits of volatile chemical detection of a parasitoid wasp, *Microplitis croceipes* and an electronic nose: A comparative study. Trans. ASAE.

[58] Zhang H., Wang J. (2007). Detection of age and insect damage incurred by wheat, with an electronic nose. J. Stored Prod. Res..

[59] Rains C.G., Tomberlin J.K., Kulasiri D. (2008). Using insect sniffing devices for detection. Trends Biotechnol..

[60] Lan Y.B., Zheng X.Z., Westbrook J.K., Lopez J., Lacey R., Hoffmann W.C. (2008). Identification of stink bugs using an electronic nose. J. Bionic Eng..

[61] Wilson A.D., Oberle C.S. (2011). Development of an electronic-nose technology for the rapid detection and discrimination of subterranean termites within wood in service. Phytopathology.

[62] Lechuga L.M., Calle A., Golmayo D., Briones F. (1991). Hydrogen sensor based on a Pt/GaAs Schottky diode. Sens. Actuator B Chem..

[63] Albert K.J., Myrick M.L., Brown S.B., Milanovich F.P., Walt D.R. (2000). High-speed fluorescence detection of explosives-like vapors. Anal. Chem..

[64] Trocino S., Donato A., Latino M., Donato N., Leonardi S.G., Neri G. (2012). Pt-TiO2/MWCNTs hybrid composites for monitoring low hydrogen concentrations in air. Sensors.

[65] Strom G., West J., Wessen B., Palmgren U., Samson R.A., Flannigan B., Flannigan M.E., Verhoefl A.P., Adan O.C.G., Hoekstra E.S. (1994). Quantitative analysis of microbial volatiles in damp Swedish houses. Health Implications of Fungi in Indoor Environments.

[66] Hwang J., Shin C., Yoe H. (2010). Study on an agricultural environment monitoring server system using wireless sensor networks. Sensors.

[67] Mothé G., Castro M., Sthel M., Lima G., Brasil L., Campos L., Rocha A., Vargas H. (2010). Detection of greenhouse gas precursors from diesel engines using electrochemical and photoacoustic sensors. Sensors.

[68] Gawas U.B., Verenkar V.M.S., Patil D.R. (2011). Nanostructured ferrite based electronic nose sensitive to ammonia at room temperature. Sci. Technol..

[69] Sato T., Breedom M., Miura N. (2012). Improvement of toluene selectivity via the application of an ethanol oxidizing catalytic cell upstream of a YSZ-based sensor for air monitoring application. Sensors.

[70] Trevathan J., Johnstone R., Chiffings T., Atkinson I., Bergmann N., Read W., Theiss S., Myers T., Stevens T. (2012). SEMAT—The next generation of inexpensive marine environmental monitoring and measurement systems. Sensors.

[71] Feng L., Musto C.J., Kemling J.W., Lim S.H., Suslick K.S. (2010). A colorimetric sensor array for identification of toxic gases below permissible exposure limits. Chem. Commun..

[72] Dighavkar C., Patil A., Patil S., Borse R. (2010). Al-doped TiO_2_ thick film resistors as H_2_S gas sensor. Sci. Technol..

[73] Fine G.F., Cavanagh L.M., Afonja A., Binions R. (2010). Metal oxide semi-conductor gas sensors in environmental monitoring. Sensors.

[74] Xie C., Xiao L., Hu M., Bai Z., Xia X., Zeng D. (2010). Fabrication and formaldehyde gas-sensing property of ZnO-MnO2 coplanar gas sensor arrays. Sens. Actuator B Chem..

[75] Ling Y.P., Heng L.Y. (2010). A potentiometric formaldehyde biosensor based on immobilization of alcohol oxidase on acryloxysuccinimide-modified acrylic microspheres. Sensors.

[76] Zhang X., Yang B., Wang X., Luo C. (2012). Effect of plasma treatment on multi-walled carbon nanotubes for the detection of H_2_S and SO_2_. Sensors.

[77] Durrani S.M.A., Al-Kuhaili M.F., Bakhtiari I.A., Haider M.B. (2012). Investigation of the carbon monoxide gas sensing characteristics of tin oxide mixed cerium oxide thin films. Sensors.

[78] Singh S., Hines E.L., Garner J.W. (1996). Fuzzy neural computing of coffee and tainted-water data from an electronic nose. Sens. Actuator B Chem..

[79] Chang C.C., Saad B., Surif M., Ahmad M.N., Shakaffm A.Y.M. (2008). Disposable etongue for the assessment of water quality in fish tanks. Sensors.

[80] O'Connor E., Smeaton A.F., O'Connor N.E., Regan F. (2012). A neural network approach to smarter sensor networks for water quality monitoring. Sensors.

[81] Labrador R., Soto J., Martinez-Manez R., Gil L. (2009). An electronic tongue for qualitative analyses of anions in natural water. J. Appl. Electrochem..

[82] Zhang H., Wang J. (2009). Evaluation of peach quality attributes using an electronic nose. Sens. Mater..

[83] Lan Y.B., Wang S.Z., Yin Y.G., Hoffmann W.C., Zheng X.Z. (2008). Using a surface plasmon resonance biosensor for rapid detection of *Salmonella typhimurium* in chicken carcass. J. Bionic Eng..

[84] Weingart O.G., Gua H., Crevoisier F., Heitger F., Avondet M.-A., Sigrist H. (2012). A bioanalytical platform for simultaneous detection and quantification of biological toxins. Sensors.

[85] Wilson A.D., Lester D.G. (1999). Utilization of aromascan analysis to identify host species of forest pathogens from woody samples. Proc. Miss. Assoc. Pl. Pathol. Nematol..

[86] Baietto M., Wilson A.D., Bassi D., Ferrini F. (2010). Evaluation of three electronic noses for detecting incipient wood decay. Sensors.

[87] Zondevan C., Muresan S., de Jonge H.G., Thoden van Velzen E.U., Wilkinson C., Nijhus H.H., Leguijt T. (1999). Controlling Maillard reactions in the heating process of blockmilk using an electronic nose. J. Agric. Food Chem..

[88] Navràtil M., Cimander C., Mandenius C.F. (2004). On-line multisensor monitoring of yogurt and Filjölk fermentations of production scale. J. Agric. Food Chem..

[89] Lorenz J.K., Reo J.P., Hendl O., Worthington J.H., Petrossian V.D. (2009). Evaluation of a taste sensor instrument (electronic tongue) for use in formulation development. Int. J. Pharm..

[90] Wilson A.D., Işin A. (2011). Future applications of electronic-nose technologies in healthcare and biomedicine. Wide Spectra of Quality Control.

[91] Pavlou A.K., Magan N., Sharp D., Brown J., Barr H., Turner A.P. (2000). An intelligent rapid odour recognition model in discrimination of *Helicobacter pylori* and other gastroesophageal isolates *in vitro*. Biosens. Bioelectron..

[92] Pavlou A., Turner A.P.F., Magan N. (2002). Recognition of anaerobic bacterial isolates *in vitro* using electronic nose technology. Lett. Appl. Microbiol..

[93] Hay P., Tummon A., Ogunfile M., Adebiyi A., Adefowora A. (2003). Evaluation of a novel diagnostic test for bacterial vaginosis: the electronic nose. Int. J. STD Aids.

[94] Moens L., van Hoeyveld E., Peetermans W.E., de Boeck C., Verhaegen J., Bossuyt X. (2006). Mannose-binding lectin genotype and invasive pneumococcal infection. Hum. Immunol..

[95] Siripatrawan U. (2008). Rapid differentiation between *E. coli* and *Salmonella typhimurium* using metal oxide sensors integrated with pattern recognition. Sens. Actuator B Chem..

[96] Hamilton S., Hepher M.J., Sommerville J. (2006). Detection of *Serpula lacrymans* infestation with a polypyrrole sensor array. Sens. Actuator B Chem..

[97] Gardner J.W., Craven M., Dow C., Hines E.L. (1998). The prediction of bacteria type and culture growth phase by an electronic nose with a multi-layer perceptron network. Meas. Sci. Technol..

[98] Hahn F. (2009). Actual pathogen detection: Sensors and algorithms—A review. Algorithms.

[99] Kuang Y., Biran I., Walt D.R. (2004). Living bacterial cell array for genotoxin monitoring. Anal. Chem..

[100] Moyo M., Okonkwo J.O., Agyei N.M. (2012). Recent advances in polymeric materials used as electron mediators and immobilizing matrices in developing enzyme electrodes. Sensors.

[101] Yang M.-Z., Dai C.-L., Lu D.-H. (2010). Polypyrrole porous micro humidity sensor integrated with a ring oscillator circuit on chip. Sensors.

[102] Nizhnik O., Higuchi K., Maenaka K. (2012). Self-calibrated humidity sensor in CMOS without post-processing. Sensors.

[103] Liao P.-J., Chang J.-S., Chao S.D., Chang H.-C., Huang K.-R., Wu K.-C., Wung T.-S. (2010). A combined experimental and theoretical study on the immunoassay of human immunoglobulin using a quartz crystal microbalance. Sensors.

[104] Ast C., Schmälzlin E., Löhmannsröben H.-G., van Dongen J.T. (2012). Optical oxygen micro- and nanosensors for plant application. Sensors.

[105] Baldwin E., Plotto A., Manthey J., McCollum G., Bai J., Irey M., Cameron R., Luzio G. (2010). Effect of *Liberibacter* infection (Huanglongbing disease) of citrus on orange fruit physiology and fruit/fruit juice quality: chemical and physical analyses. J. Agric. Food Chem..

[106] Wilson A.D., Lester D.G. (1997). Use of an electronic-nose device for profiling headspace volatile metabolites to rapidly identify phytopathogenic microbes. Phytopathology.

[107] Wilson A.D., Lester D.G. (1998). Application of aromascan analysis to detect and diagnose oak wilt in live oaks. Phytopathology.

[108] Prithiviraj B., Vikram A., Kushalappa A.C., Yaylayan V. (2004). Volatile metabolite profiling for the discrimination of onion bulbs infected by *Erwinia carotovora* spp. *carotovora*, *Fusarium oxysporum*, and *Botrytis allii*. Eur. J. Plant Pathol..

[109] Vikram A., Hamzehzarghani H., Kushalappa A.C. (2005). Volatile metabolites from the headspace of onion bulbs inoculated with postharvest pathogens as a tool for disease discrimination. Can. J. Plant Pathol..

[110] Laothawornkitkul J., Moore J.P., Taylor J.E., Possell M., Gibson T.D., Hewitt C.N., Paul N.D. (2008). Discrimination of plant volatile signatures by an electronic nose: A potential technology for plant pest and disease monitoring. Environ. Sci. Technol..

[111] Jansen R.M.C., Wildt J., Kappers I.F., Bouwmeester H.J., Hofstee J.W., van Henten E.J. (2011). Detection of diseased plants by analysis of volatile organic compound emission. Annu. Rev. Phytopathol..

[112] Spinelli F., Cellini A., Vanneste J.L., Rodriquez-Estrada M.T., Costa G., Savioli S., Harren F.J.M., Cristescu S.M. (2012). Emission of volatile compounds by *Erwinia amylovora*: Biological activity in vitro and possible exploitation for bacterial identification. Trees.

[113] Gao Y., Jin Y., Li H., Chen H. (2005). Volatile organic compounds and their roles in bacteriostasis in five conifer species. J. Integr. Plant Biol..

[114] Magan N., Evans P. (2000). Volatiles as an indicator of fungal activity and differentiation between species, and the potential use of electronic nose technology for early detection of grain spoilage. J. Stored Prod. Res..

[115] De Lacy Costello B.J.P., Ewen R.J., Gunson H.E., Ratcliffe N.M., Spencer-Phillips P.T.N. (2002). Sensors for Early Warning of Postharvest Spoilage in Potato Tubers.

[116] Li Z., Wang N., Vijaya Raghavan G.S., Vigneault C. (2009). Ripeness and rot evaluation of “Tommy Atkins” mango fruit through volatiles detection. J. Food Eng..

[117] Li C., Krewer G.W., Ji P., Scherm H., Kays S.J. (2010). Gas sensor array for blueberry fruit disease detection and classification. Postharvest Biol. Technol..

[118] Santonico M., Bellincontro A., de Santis D., di Natale C., Mencarelli F. (2010). Electronic nose to study postharvest dehydration of wine grapes. Food Chem..

[119] Ewen R.J., Jones P.R.H., Ratcliffe N.M., Spencer-Phillips P.T.N. (2004). Identification by gas chromatography-mass spectrometry of the volatile organic compounds emitted from the woodrotting fungi *Serpula lacrimans* and *Coniophora puteana*, and from *Pinus sylvestris* timber. Mycol. Res..

[120] Baietto M. (2008). Development of a New Non-Invasive Tool for the Assessment of Decays in the Urban Environment. Ph.D. Thesis.

[121] Nylander C., Armgarth M., Lundström I. (1983). An ammonia detector based on a conducting polymer. Anal. Chem. Symp. Ser..

[122] Winquist F., Spetz A., Armgarth M., Lundström I., Danielsson B. (1985). Biosensors based on ammonia sensitive metal-oxide-semiconductor structures. Sens. Actuator B Chem..

[123] Persaud K.C., Payne P.A., Khaffaf S.M., Dowdeswell R.M., Hobbs P.J., Misselbrook T.H., Sneath R.W. Application of conducting polymer odor sensing arrays to agricultural malodour monitoring.

[124] McGinley M.A., McGinley C.M. Measuring composting odors for decision making.

[125] Sohn J.H., Dunlop M., Hudson N., Kim T.I., Yoo Y.H. (2009). Non-specific conducting polymer-based array capable of monitoring odour emissions from a biofiltration system in a piggery building. Sens. Actuator B Chem..

[126] Wilson A.D. Application of a conductive polymer electronic-nose device to identify aged woody samples.

[127] Mielle P. (1996). Electronic noses: Towards the objective instrumental characterization of food aroma. Trends Food Sci. Technol..

[128] Hruskar M., Major N., Krpan M. (2010). Application of a potentiometric sensor array as a technique in sensory analysis. Talanta.

[129] Zhang H., Wang J., Sheng Y. (2008). Predictions of acidity, soluble solids and firmness of pear using electronic nose technique. J. Food Eng..

[130] Casilli S., de Luca M., Apetrei C., Parra V., Arrieta Á.A., Valli L., Jiang J., Rodríguez-Méndez M.L., de Saja J.A. (2005). Langmuir-Blodgett and Langmuir-Schaefer films of homoleptic and heteroleptic phthalocyanine complexes as voltammetric sensors: Applications to the study of antioxidants. Appl. Surf. Sci..

[131] Cosio M.S., Buratti S., Mannino S., Benedetti S. (2006). Use of an electrochemical method to evaluate the antioxidant activity of herb extracts from the Labiatae family. Food Chem..

[132] Li W., Hosseinian F.S., Tsopmo A., Friel J., Beta T. (2009). Evaluation of antioxidant capacity and aroma quality of breast milk. Nutrition.

[133] Li W., Friel J., Beta T. (2010). An evaluation of the antioxidant properties and aroma quality of infant cereals. Food Chem..

[134] Apetrei C., Rodríguez-Méndez M.L., Parra V., Gutierrez F., de Saja J.A. (2004). Array of voltammetric sensors for the discrimination of bitter solutions. Sens. Actuator B Chem..

[135] Thorngate J.H., Noble A.C. (1995). Sensory evaluation of bitterness and astringency of 3*R*(-)-epicatechin and 3*S*(+)-catechin. J. Sci. Food Agric..

[136] Kaneda H., Watari J., Takashio M. (2003). Measuring astringency of beverages using a quartz-crystal microbalance. J. Am. Soc. Brew. Chem..

[137] Legin A., Rudnitskaya A., Kirsanov D., Frolova Yu., Clapham D., Caricofe R. Assessment of bitterness intensity and suppression effects using an electronic tongue.

[138] Apetrei C., Apetrei I.M., Villanueva S., de Saja J.A., Gutierrez-Rosales F., Rodriguez-Mendez M.L. (2010). Combination of an e-nose, an e-tongue and an e-eye for the characterisation of olive oils with different degree of bitterness. Anal. Chim. Acta.

[139] Arrieta A.A., Apetrei C., Rodríguez-Méndez M.L., de Saja J.A. (2004). Voltammetric sensor array based on conducting polymer-modified electrodes for the discrimination of liquids. Electrochim. Acta.

[140] Zhang C., Bailey D.P., Suslick K.S. (2006). Colorimetric sensor arrays for the analysis of beers: A feasibility study. J. Agric. Food Chem..

[141] Concina I., Falasconi M., Gobbi E., Bianchi F., Musci M., Mattarozzi M., Pardo M., Mangia A., Careri M., Sbeveglieri G. (2009). Early detection of microbial contamination in processed tomato by electronic nose. Food Control.

[142] Rudnitskaya A., Polshin E., Kirsanov D., Lammertyn J., Nicolai B., Saison D., Delvaux F.R., Delvaux F., Legin A. (2009). Instrumental measurement of beer taste attributes using an electronic tongue. Anal. Chim. Acta.

[143] Polshin E., Rudnitskaya A., Kirsanov D., Legin A., Saison D., Delvaux F., Delvaux F.R., Nicolaï B.M., Lammertyn J. (2010). Electronic tongue as a screening tool for rapid analysis of beer. Talanta.

[144] Brown R.J.C., Keates A.C., Brewer P.J. (2010). Sensitivities of a standard test method for the determination of the pHe of bioethanol and suggestions for improvement. Sensors.

[145] Yang Z., Dong F., Shimizu K., Kinoshita T., Kanamori M., Morita A., Watanabe N. (2009). Identification of coumarin-enriched Japanese green teas and their particular flavor using electronic nose. J. Food Eng..

[146] Medeiros E.S., Gregorio R., Martinez R.A., Mattoso L.H.C. (2009). A taste sensor array based on polyaniline nanofibers for orange juice quality assessment. Sens. Lett..

[147] Rudnitskaya A., Rocha S.M., Legin A., Pereira V., Marques J.C. (2010). Evaluation of the feasibility of the electronic tongue as a rapid analytical tool for wine age prediction and quantification of the organic acids and phenolic compounds. The case-study of Madeira wine. Anal. Chim. Acta.

[148] Labrador R.H., Masot R., Alcañiz M., Baigts D., Soto J., Martínez-Mañez R., García-Breijo E., Gil L., Barat J.M. (2010). Prediction of NaCl, nitrate and nitrite contents in minced meat by using a voltammetric electronic tongue and an impedimetric sensor. Food Chem..

[149] Chen Q., Zhao J., Guo Z., Wang X. (2010). Determination of caffeine content and main catechins contents in green tea (*Camellia sinensis* L.) using taste sensor technique and multivariate calibration. J. Food Compos. Anal..

[150] Holscher W., Vitzthum O.G., Steinhart H. (1990). Identification and sensorial evaluation of aroma impact-compounds in roasted Colombian coffee. Café Cacao Thé.

[151] Holscher W., Vitzthum O.G., Steinhart H. (1992). Prenyl alcohol—Source for odorants in roasted coffee. J. Agric. Food Chem..

[152] Grosch W. (1998). Flavour of coffee. Food.

[153] Lindinger C., Labbe D., Pollien P., Rytz A., Juillerat M.A., Yeretzian C., Blank I. (2008). When machine tastes coffee: Instrumental approach to predict the sensory profile of espresso coffee. Anal. Chem..

[154] Hartman J.D. (1954). A possible method for the rapid estimation of flavours in vegetables. Proc. Am. Soc. Hort. Sci..

[155] Ulmer H., Mitrovics J., Noetzel G., Wiemar U., Gopel W. (1992). Odours and flavours identified with hybrid modular sensor systems. Sens. Actuator B Chem..

[156] Gardner J.W., Pearce T.C., Friel S., Bartlett P.N., Blair N.A. (1994). Multisensor system for beer flavour monitoring using an array of conducting polymers and predictive classifiers. Sens. Actuator B Chem..

[157] Strassburger K.J. (1996). Electronic nose evaluation in the flavor industry: It really works!. Food Test. Anal..

[158] Irmler S., Heusler M.L., Raboud S., Schlichtherle-Cerny H., Casey M.G., Eugster-Meier E. (2006). Rapid volatile metabolite profiling of *Lactobacillus casei* strains: Selection of flavour producing cultures. Aust. J. Dairy Technol..

[159] Beullens K., Meszaros P., Vermeir S., Kirsanov D., Legin A., Buysens S., Cap N., Nicolai B.M., Lammertyn J. (2008). Analysis of tomato taste using two types of electronic tongues. Sens. Actuator B Chem..

[160] Sun H., Mo Z.H., Choy J.T.S., Zhu D.R., Fung Y.S. (2008). Piezoelectric quartz crystal sensor for sensing taste-causing compounds in food. Sens. Actuator B Chem..

[161] Wang B., Xu S., Sun D.W. (2010). Application of the electronic nose to the identification of different milk flavorings. Food Res. Int..

[162] García-Martínez T., Bellincontro A., de Lerma M.D.L.N.L., Peinado R.A., Mauricio J.C., Mencarelli F., Moreno J.J. (2011). Discrimination of sweet wines partially fermented by two osmo-ethanol-tolerant yeasts by gas chromatographic analysis and electronic nose. Food Chem..

[163] Calkin R.R., Jellinek J.S. (1994). Perfumery: Practice and Principles.

[164] Hanaki S., Nakamoto T., Moriizumi T. (1996). Artificial odor recognition system using neural network for estimating sensory quantities of blended fragrance. Sens. Actuator B Chem..

[165] Anselmi C., Centini M., Fedeli P., Paoli M.L., Sega A., Scesa C., Pelosi P. (2000). Unsaturated hydrocarbons with fruity and floral odors. J. Agric. Food Chem..

[166] Maul F., Sargent S.A., Balaban M.O., Baldwin E.A., Huber D.J., Sims C.A. (1998). Aroma volatile profiles from ripe tomatoes are influenced by physiological maturity at harvest: An application for electronic nose technology. J. Am. Soc. Hort. Sci..

[167] Supriyadi S., Shimuzu K., Suzuky M., Yoshida K., Muto T., Fujita A., Tomita N., Watanabe N. (2004). Maturity discrimination of snake fruit (*Salacca edulis* Reinw.) cv. Pondoh based on volatiles analysis using an electronic nose device equipped with a sensor array and fingerprint mass spectrometry. Flavour Fragr. J..

[168] Falasconi M., Pardo M., Sberveglieri G., Riccò I., Bresciani A. (2005). The novel EOS835 electronic nose and data analysis for evaluating coffee ripening. Sens. Actuator B Chem..

[169] Pathange L.P., Mallikarjunan P., Marini R.P., O'Keefe S., Vaughan D. (2006). Non-destructive evaluation of apple maturity using an electronic nose system. J. Food Eng..

[170] Gòmez A.H., Hu G., Wang J., Pereira A.G. (2006). Evaluation of tomato maturity by electronic nose. Comput. Electron. Agric..

[171] Lebrun M., Plotto A., Goodner K., Ducamp M.N., Baldwin E. (2008). Discrimination of mango fruit maturity by volatiles using the electronic nose and gas chromatography. Postharvest Biol. Technol..

[172] Du X., Bai J., Plotto A., Baldwin E.A., Whitaker V., Souseff R. (2010). Electronic nose for detecting strawberry fruit maturity. Proc. Fla. State Hort..

[173] Pani P., Leva A.A., Riva M., Maestrelli A., Torreggiani D. (2008). Influence of an osmotic pre-treatment on structure-property relationships of air-dehydrated tomato slices. J. Food Eng..

[174] Defilippi B.G., Juan W.S., Valdés H., Moya-León M.A., Infante R., Campos-Vargas R. (2009). The aroma development during storage of Castlebrite apricots as evaluated by gas chromatography, electronic nose, and sensory analysis. Postharvest Biol. Tecnol..

[175] Söderström C., Borén H., Winquist F., Krantz-Rülcker C. (2003). Use of an electronic tongue to analyze mold growth in liquid media. Int. J. Food Microbiol..

[176] Perkowski J., Buśko M., Chmielewski J., Góral T., Tyrakowska B. (2008). Content of trichodiene and analysis of fungal volatiles (electronic nose) in wheat and triticale grain naturally infected and inoculated with *Fusarium culmorum*. Int. J. Food Microbiol..

[177] Pisanelli A.M., Qutob A.A., Travers P., Szyszko S., Persaud K.C. (1994). Applications of multi-array polymer sensors to food industries. Life Chem. Rep..

[178] Persaud K.C., Qutob A.A., Travers P., Pisanelli A.M., Szyszko S., Kurihara K., Suzuki N., Ogawa H. (1994). Odor evaluation of foods using conducting polymer arrays and neural net pattern recognition. Olfaction and Taste XI.

[179] Bartlett P.N., Elliott J.M., Gardner J.W. (1997). Electronic noses and their applications in the food industry. Food Technol..

[180] Schaller E., Bosset J.O., Esher F. (1998). Electronic noses and their application to food. LWT Food Sci. Technol..

[181] Deisingh A.K., Stone D.C., Thompson M. (2004). Applications of electronic noses and tongues in food analysis. Int. J. Food Sci. Technol..

[182] Berrueta L.A., Alonso-Salces R.M., Heberger K. (2007). Supervised pattern recognition in food analysis. J. Chromatogr. A.

[183] Scampicchio M., Ballabio D., Arecchi A., Cosio S.M., Mannino S. (2008). Amperometric electronic tongue for food analysis. Microchim. Acta.

[184] Peres A., Dias L., Barcelos T., Sa Morais J., Machado A. (2009). An electronic tongue for juice level evaluation in non-alcoholic beverages. Proc. Chem..

[185] Dias L.G., Peres A.M., Barcelos T.P., Sá Morais J., Machado A.A.S.C. (2011). Semi-quantitative and quantitative analysis of soft drinks using an electronic tongue. Sens. Actuator B Chem..

[186] Iiyama S., Ezaki S., Toko K. (2009). Sensitivity-improvement of taste sensor by change of lipid concentration in membrane. Sens. Actuator B Chem..

[187] Berdagué J.L., Talou T. (1993). Examples of applications for meat products of semiconductor gas sensors. Sci. Aliments.

[188] Grigioni G.M., Margaría C.A., Pensel N.A., Sánchez G., Vaudagna S.R. (2000). Warmed-over flavour analysis in low temperature-long time processed meat by an “electronic nose”. Meat Sci..

[189] Russell P. (1995). Sensory analysis. Milk Ind..

[190] Romero G., Díaz J.R., Sabater J.M., Perez C. (2012). Evaluation of commercial probes for on-line electrical conductivity measurements during goat gland milking process. Sensors.

[191] Lerma-Garcia M.J., Simo-Alfonso E.F., Bendini A., Cerretani L. (2009). Metal oxide semiconductor sensors for monitoring of oxidative status evolution and sensory analysis of virgin olive oils with different phenolic content. Food Chem..

[192] Mildner-Szkudlarz S., Jelen H.H. (2010). Detection of olive oil adulteration with rapeseed and sunflower oils using MOS electronic nose and SMPE-MS. J. Food Qual..

[193] Zhang C., Suslick K.S. (2007). Colorimetric sensor array for soft drink analysis. J. Agric. Food Chem..

[194] Ohata M., Tominaga T., Dubourdieu D., Kubota K., Sugawara E. (2009). Quantification and odor contribution of 2-furanmethanethiol in different types of fermented soybean paste miso. J. Agric. Food Chem..

[195] Zhang H., Balaban M., Portier K., Sims C.A. (2005). Quantification of spice mixture compositions by electronic nose: Part II. Comparison with GC and sensory methods. J. Food Sci..

[196] Rajamäki T., Alatomi H., Titvanen T., Skyttä E., Smolander M., Ahvenainen R. (2004). Application of an electronic nose for quality assessment of modified atmosphere packaged poultry meat. Food Control.

[197] Echeverria G., Graell J., Lopez M.L., Brezmes J., Correig X. (2005). Volatile production in “Fuji” apples stored under different atmospheres measured by headspace/gas chromatography and electronic nose. Acta Hort..

[198] Arrieta A., Rodriguez-Mendez M.L., de Saja J.A. (2003). Langmuir-Blodgett film and carbon paste electrodes based on phthalocyanines as sensing units for taste. Sens. Actuator B Chem..

[199] Daillant-Spinnler B., MacFie H.J.H., Beyts P.K., Hedderley D. (1996). Relationships between perceived sensory properties and major preference directions of 12 varieties of apples from the Southern Hemisphere. Food Qual. Prefer..

[200] Bartoshuk L.M. (2000). Comparing sensory experiences across individuals: Recent psychophysical advances illuminate genetic variation in taste perception. Chem. Sens..

[201] Bleibaum R.N., Stone H., Tan T., Labreche S., Saint-Martin E., Isz S. (2002). Comparison of sensory and consumer results with electronic nose and tongue sensors for apple juices. Food Qual. Prefer..

[202] Hayasaka Y., Baldock G.A., Pollnitz A.P. (2005). Contributions of mass spectrometry in The Australian Wine Research Institute to advances in knowledge of grape and wine constituents. Aust. J. Grape Wine Res..

[203] Falcão L.D., de Revel G., Rosier J.P., Bordignon-Luiz M.T. (2008). Aroma impact components of Brazilian Cabernet Sauvignon wines using detection frequency analysis (GC-olfactometry). Food Chem..

[204] Vera L., Mestres M., Boqué R., Busto O., Guasch J. (2010). Use of synthetic wine for models transfer in wine analysis by HS-MS e-nose. Sens. Actuator B Chem..

[205] Santos J.P., Lozano J., Aleixandre M., Arroyo T., Cabellos J.M., Gil M., Horrillo M.D.C. (2010). Threshold detection of aromatic compounds in wine with an electronic nose and a human sensory panel. Talanta.

[206] Aguilera T., Lozano J., Paredes J.A., Álvarez F.J., Suárez J.I. (2012). Electronic nose based on independent component analysis combined with partial least squares and artificial neural networks for wine prediction. Sensors.

[207] Shurmer H.V., Gardner J.W., Chan H.T. (1989). The application of discrimination techniques to alcohols and tobacco using tin oxide sensors. Sens. Actuator B Chem..

[208] Aishima T. (1991). Discrimination of liqueur aromas by pattern recognition analysis of responses from a gas sensor array. Anal. Chim. Acta.

[209] Solís-Solís H.M., Calderón-Santoyo M., Gutierrez-Martinez P., Schorr-Galindo S., Ragazzo-Sánchez J. (2007). Discrimination of eight varieties of apricot (*Prunus armeniaca* L.) by electronic nose LLE and SPME using GC-MS and multivariate analysis. Sens. Actuator B Chem..

[210] Gatti E., Defilippi B.G., Predieri S., Infante R. (2009). Apricot (*Prunus armeniaca* L.) quality and breeding perspectives. J. Food Agric. Environ..

[211] Ponzoni A., Depari A., Falasconi M., Comini E., Flammini A., Marioli D., Taroni A., Sberveglieri G. (2008). Bread baking aromas detection by low-cost electronic nose. Sens. Actuator B Chem..

[212] Wu R.J., Yeh C.H., Yu M.R., Chen H.W. (2008). Application of taste sensor array to sports drinks by using impedance measurement technology. Sens. Lett..

[213] Rodriguez-Mendez M.L., Apetrei C., de Saja J.A. (2008). Evaluation of the polyphenolic content of extra virgin olive oils using an array of voltammetric sensors. Electrochim. Acta.

[214] Ciosek P., Kraszewska Z., Wroblenski W. (2009). Polyurethane membranes used in integrated electronic tongue for the recognition of tea and herbal products. Electroanalysis.

[215] Ding F., Liu B., Deng X., Wang Z., Xie Z., Fang Y., Xu J. (2010). Delayed bitterness of six sweet oranges (*Citrus sinensis* Osbeck). J. Huazhong Agric. Univ..

[216] Li Z., Vijaya Raghavan G.S., Wang N. (2009). Carrot volatiles monitoring and control in microwave drying. LWT Food Sci. Technol..

[217] Gursoy O., Somervuo P., Alatossava T. (2009). Preliminary study of ion mobility based electronic nose MGD-1 for discrimination of hard cheeses. J. Food Eng..

[218] Pais V.F., Oliveira J.A.B.P., Gomes M.T.S.R. (2012). An electronic nose based on coated piezoelectric quartz crystals to certify ewes' cheese and to discriminate between cheese varieties. Sensors.

[219] Ravi R., Harte J.B. (2009). Milling and physicochemical properties of chickpea (*Cicer arietinum* L.) varieties. J. Sci. Food Agric..

[220] Goodner K., Baldwin E.A., Jordan M., Shaw P.E. (2002). The comparison of an electronic nose and gas chromatograph of differentiating NFC orange juices. Proc. Fla. State Hort..

[221] Reinhard H., Sager F., Zoller O. (2008). Citrus juice classification by SPME-GC-MS and electronic nose measurements. LWT Food Sci. Technol..

[222] Gardner J.W., Shurmer H.V., Tan T.T. (1992). Application of an artificial electronic nose to the discrimination of coffee. Sens. Actuator B Chem..

[223] Suslick B.A., Feng L., Suslick K.S. (2010). Discrimination of complex mixtures by a colorimetric sensor array: Coffee aromas. Anal. Chem..

[224] Rodriguez J., Duran C., Reyes A. (2010). Electronic nose for quality control of Colombian coffee through the detection of defects in “Cup Tests”. Sensors.

[225] Oliveri P., Baldo M.A., Daniele S., Forina M. (2009). Development of a voltammetric electronic tongue for discrimination of edible oils. Anal. Bioanal. Chem..

[226] Van Ruth S.M., Rozijn M., Koot A., Garcia R.P., van der Kamp H., Codony R. (2010). Authentication of feeding fats: Classification of animal fats, fish oils and recycled cooking oils. Anim. Feed Sci. Technol..

[227] Cano M., Roales J., Castillero P., Mendoza P., Calero A.M., Jiménez-Ot C., Pedrosa J.M. (2011). Improving the training and data processing of an electronic olfactory system for the classification of virgin olive oil into quality categories. Sens. Actuator B Chem..

[228] Hong E.J., Park S.J., Choi J.Y., Noh B.S. (2011). Discrimination of palm olein oil and palm stearin oil mixtures using a mass spectrometry based electronic nose. Food Sci. Biotechnol..

[229] Vernat-Rossi V., Vernat G., Berdagué J.L. (1996). Discrimination of agroalimentary products by gas sensors with semiconductors functioning with ambient air of the laboratory. Various approaches of signal treatment. Analysis.

[230] Jonsson A., Winquist F., Schnürer J., Sundgren H., Lundtröm I. (1997). Electronic nose for microbial quality classification of grains. Int. J. Food Microbiol..

[231] Wei Z., Wang J., Liao W. (2009). Technique potential for classification of honey by electronic tongue. J. Food Eng..

[232] Zakaria A., Shakaff A.Y., Masnan M.J., Ahmad M.N., Adom A.H., Jaafar M.N., Ghani S.A., Abdullah A.H., Aziz A.H.A., Kamarudin L.M. (2011). A biomimetic sensor for the classification of honeys of different floral origin and the detection of adulteration. Sensors.

[233] Ciosek P., Wroblewski W. (2008). Miniaturized electronic tongue with an integrated reference microelectrode for the recognition of milk samples. Talanta.

[234] Men H., Ge Z., Guo Y., An L., Peng Y. Biomimetic electronic tongue for classification of mineral water.

[235] Sghaier K., Barhoumi H., Maaref A., Siadat M., Jaffrezic-Renault N. (2009). Classification and discrimination of different Tunisian water samples using an electronic tongue. Sens. Lett..

[236] Oshita S., Shima K., Haruta T., Seo Y., Kawagoe Y., Nakayama S., Takahara H. (2000). Discrimination of odors emanating from “La France” pear by semi-conducting polymer sensors. Comput. Electron. Agric..

[237] Zheng X.Z., Lan Y.B., Zhu J.M., Westbrook J., Hoffmann W.C., Lacey R.E. (2009). Rapid identification of rice samples using an electronic nose. J. Bionic Eng..

[238] Gasso-Tortajada V., Ward A.J., Mansur H., Brøchner T., Sørensen C.G., Green O. (2010). A novel acoustic sensor approach to classify seeds based on sound absorption spectra. Sensors.

[239] Palit M., Tudu B., Dutta P.K., Dutta A., Jana A., Roy J.K., Bhattacharyya N. (2009). Classification of black tea taste and correlation with tea taster's mark using voltammetric electronic tongue. IEEE Trans. Instrum. Meas..

[240] Xiao H., Wang J. (2009). Discrimination of Xihulongjing tea grade using an electronic tongue. Afr. J. Biotechnol..

[241] Scarpa A., Bernardi S., Fachechi L., Olimpico F., Passamano M., Greco S. Polypyrrole polymers used for 2,4,6-trichloroanisole discrimination in cork stoppers by LibraNose.

[242] McKellar R.C., Vasantha Rupasinghe H.P., Lu X., Knight K.P. (2005). The electronic nose as a tool for the classification of fruit and grape wines from different Ontario wineries. J. Sci. Food Agric..

[243] Fu J., Huang C., Xing J., Zheng J. (2012). Pattern classification using an olfactory model with PCA feature selection in electronic noses: Study and application. Sensors.

[244] Parra V., Arrieta A.A., Fernandez-Escudero J.A., Garcia H., Apetrei C., Rodriguez-Mendez M.L., Saja J.A. (2006). E-tongue based on a hybrid array of voltammetric sensors based on phthalocyanines, perylene derivatives and conducting polymers: Discrimination capability towards red wines elaborated with different varieties of grapes. Sens. Actuator B Chem..

[245] Lozano J., Santos J.P., Carmen Horrillo M. (2008). Enrichment sampling methods for wine discrimination with gas sensors. J. Food Compost. Anal..

[246] Cynkar W., Dambergs R., Smith P., Cozzolino D. (2010). Classification of Tempranillo wines according to geographic origin: Combination of mass spectrometry based electronic nose and chemometrics. Anal. Chim. Acta.

[247] Musto C.J., Lim S.H., Suslick K.S. (2009). Colorimetric detection and identification of natural and artificial sweeteners. Anal. Chem..

[248] Jonsdottir R., Olafsdottir G., Martinsdottir E., Stefansson G. (2004). Flavor characterization of ripened cod roe by gas chromatography; sensory analysis; and electronic nose. J. Agric. Food Chem..

[249] Trihaas J., Nielsen V. (2005). Electronic nose technology in quality assessment: Monitoring the ripening process of Danish blue cheese. J. Food Sci..

[250] Biolatto A., Grigioni G., Irurueta M., Rancho A.M., Taverna M., Pensel N. (2007). Seasonal variation in the odour characteristics of whole milk powder. Food Chem..

[251] Vestergaard J.S., Martens M., Turkki P. (2007). Analysis of sensory quality changes during storage of a modified atmosphere packaged meat product (pizza topping) by an electronic nose system. LWT Food Sci. Technol..

[252] Vestergaard J.S., Martens M., Turkky P. (2007). Application of an electronic nose system for prediction of sensory quality changes of a meat product (pizza topping) during storage. LWT Food Sci. Technol..

[253] Cacic F., Primorac L., Kenjeric D., Benedetti S., Madic M.L. (2009). Application of electronic nose in honey geographical origin characterization. J. Cent. Eur. Agric..

[254] Casale M., Casolino C., Oliveri P., Forina M. (2010). The potential of coupling information using three analytical techniques for identifying the geographical origin of Liguria extra virgin olive oil. Food Chem..

[255] Berna A.Z., Trowell S., Clifford D., Cynkar W., Cozzolino D. (2009). Geographical origin of Sauvignon Blanc wines predicted by mass spectrometry and metal oxide based electronic nose. Anal. Chim. Acta.

[256] He W., Hu X., Zhao L., Liao X., Zhang Y., Zhang M., Wu J. (2009). Evaluation of Chinese tea by the electronic tongue: Correlation with sensory properties and classification according to geographical origin and grade level. Food Res. Int..

[257] Paixao T.R.L.C., Bertotti M. (2009). Fabrication of disposable voltammetric electronic tongues by using Prussian blue films electrodeposited onto CD-R gold surfaces and recognition of milk adulteration. Sens. Actuator B Chem..

[258] Dias L.A., Peres A.M., Veloso A.C.A., Reis F.S., Vilas-Boas M., Machado A.A.S.C. (2009). An electronic tongue taste evaluation: Identification of goat milk adulteration with bovine milk. Sens. Actuator B Chem..

[259] Marina A.M., Man Y.B.C., Amin I. (2010). Use of the SAW sensor electronic nose for detecting the adulteration of virgin coconut oil with RBD palm kernel olein. J. Am. Oil Chem. Soc..

[260] Hilding-Ohlsson A., Fauerbach J.A., Sacco N.J., Bonetto M.C., Cortón E. (2012). Voltamperometric discrimination of urea and melamine adulterated skimmed milk powder. Sensors.

[261] Ahn S., Walt D.R. (2005). Detection of *Salmonella* spp. using microsphere-based, fiber-optic DNA microarrays. Anal. Chem..

[262] Balasubramanian S., Panigrahi S., Logue C.M., Doetkott C., Marchello M., Sherwood J.S. (2008). Independent component analysis-processed electronic nose data for predicting *Salmonella typhimurium* populations in contaminated beef. Food Control.

[263] Wang Y., Ye Z., Ying Y. (2012). New trends in impedimetric biosensors for the detection of foodborne pathogenic bacteria. Sensors.

[264] Winquist F., Sundgren H., Lundstrom I. A practical use of electronic nose: quality estimation of cod fillet bought over the counter.

[265] Olafsdottir G., Chanie E., Westad F., Jonsdottir R., Thalmann C.R., Bazzo S., Labreche S., Marcq P., Lunby F., Haugen J.E. (2005). Prediction of microbial and sensory quality of cold smoked Atlantic salmon (*Salmo salar*) by electronic nose. J. Food Sci..

[266] Olafsdottir G., Jonsdottir R., Lauzon H.L., Luten J., Kristbergsson K. (2005). Characterization of volatile compounds in chilled cod (*Gadus morhua*) fillets by gas chromatography and detection of quality indicators by an electronic nose. J. Agric. Food Chem..

[267] Haugen J.E., Chanie E., Westad F., Jonsdottir R., Bazzo S., Labreche S., Marcq P., Lundby F., Olafsdottir G. (2005). Rapid control of smoked Atlantic salmon (*Salmo salar*) quality by electronic nose: correlation with classical evaluation methods. Sens. Actuator B Chem..

[268] Barat J.M., Gil L., Garcia-Brejio E., Aristory M., Toldra F., Martinez-manez R., Soto J. (2008). Freshness monitoring of sea bream (*Sparus aurata*) with a potentiometric sensor. Food Chem..

[269] Peris M., Escuder-Gilabert L. (2009). A 21st century technique for food control: Electronic noses. Anal. Chim. Acta.

[270] Ghasemi-Varnamkhasti M., Mohtasebi S.S., Siadat M. (2010). Biomimetic-based odor and taste sensing systems to food quality and safety characterization: An overview on basic principles and recent achievements. J. Food Eng..

[271] Bai J., Baldwin E.A., Soliva Fortuny R.C., Mattheis J.P., Stanley R., Perera C., Brecht J.K. (2004). Effect of pretreatment of intact “Gala” apple with ethanol vapor, heat, or 1-methylcyclopropene on quality and shelf life of fresh-cut slices. J. Am. Soc. Hort. Sci..

[272] Plotto A., Baldwin E., McCollum G., Manthey J., Narciso J., Irey M. (2010). Effect of *Liberibacter* infection (Huanglongbing or “Greening” disease) of citrus on orange juice flavor quality by sensory evaluation. J. Food Sci..

[273] Li C., Heinemann P., Sherry R. (2007). Neural network and Bayesian network fusion models to fuse electronic nose and surface acoustic wave sensor data for apple defect detection. Sens. Actuator B Chem..

[274] Winquist F., Hornsten E.G., Sundgren H., Lundstrom I. (1993). Performance of an electronic nose for quality estimation of ground meat. Meas. Sci. Technol..

[275] Ghasemi-Varnamkhasti M., Mohtasebi S.S., Siadat M., Balasubramanian S. (2009). Meat quality assessment by electronic nose (machine olfaction technology). Sensors.

[276] Sangam V.G., Sandesh M., Krishna S., Mahadevanna S. (2010). Design of simple instrumentation for the quality analysis of milk (casein analysis). Sci. Technol..

[277] Shen N., Moizuddin S., Wilson L., Duvick S., White P., Pollak L. (2001). Relationship of electronic nose analyses and sensory evaluation of vegetable oils during storage. J. Am. Oil Chem. Soc..

[278] Ragazzo-Sanchez J.A., Chalier P., Chevalier-Lucia D., Calderon-Santoyo M., Ghommidh C. (2009). Off-flavours detection in alcoholic beverages by electronic nose coupled to GC. Sens. Actuator B Chem..

[279] Fujita A., Isogai A., Endo M., Utsunomiya H., Nakano S., Iwata H. (2010). Effects of sulfur dioxide on formation of fishy off-odor and undesirable taste in wine consumed with seafood. J. Agric. Food Chem..

[280] Simon J.E., Hetzroni A., Bordelon B., Miles G.E., Charles D.J. (1996). Electronic sensing of aromatic volatiles for quality sorting of blueberries. J. Food Sci..

[281] Costa G., Noferini M., Montefiori M., Brigati S. (2003). Non-destructive assessment methods of kiwifruit quality. Acta Hort..

[282] Benedetti S., Toppino P.M., Riva M. (2002). Study of the shelf life of manufactured Taleggio cheese: 2. Applications of the electronic nose. Sci. Technol. Lattiero Casearia.

[283] Riva M., Benedetti S., Mannino S. (2002). Shelf life of fresh cut vegetables as measured by an electronic nose: Preliminary study. Ital. Food Sci..

[284] Riva M., Benedetti S., Sinelli N. (2004). Combined techniques of NIRS and Electronic Nose for the study of the shelf life of lattiero-caseari products. Ingred. Aliment..

[285] Niruntasuk K., Innawong B., Parakulsulsatid P. Shelf life determination of vacuum fried mango chips using electronic nose.

[286] Labreche S., Bazzo S., Cade S., Chanie E. (2005). Shelf life determination by electronic nose: Application to milk. Sens. Actuat or B Chem..

[287] Chantarachoti J., Oliveira A.C.M., Himelbloom B.H., Crapo C.A., McLachlan D.G. (2006). Portable electronic nose for detection of spoiling Alaska pink salmon *(Oncorhynchus gorbuscha)*. J. Food Sci..

[288] Casalinuovo I.A., di Pierro D., Coletta M., di Francesco P. (2006). Application of electronic noses for disease diagnosis and food spoilage detection. Sensors.

[289] Gómez A.H., Wang J., Hu G., García Pereira A. (2008). Monitoring storage shelf life of tomato using electronic nose technique. J. Food Eng..

[290] Wang Y., Wang J., Zhou B., Lu Q. (2009). Monitoring storage time and quality attribute of egg based on electronic nose. Anal. Chim. Acta.

[291] Limbo S., Torri L., Sinelli N., Franzetti L., Casiraghi E. (2010). Evaluation and predictive modeling of shelf life of minced beef stored in high-oxygen modified atmosphere packaging at different temperatures. Meat Sci..

[292] Torri L., Sinelli N., Limbo S. (2010). Shelf life evaluation of fresh-cut pineapple by using an electronic nose. Postharvest Biol. Tecnol..

[293] Argyri A.A., Panagou E.Z., Tarantilis P.A., Polysiou M., Nychas G.J.E. (2010). Rapid qualitative and quantitative detection of beef fillets spoilage based on Fourier transform infrared spectroscopy data and artificial neural networks. Sens. Actuat or B Chem..

[294] Gil L., Barat J.M., Garcia-Breijo E., Ibañez J., Martínez-Máñez R., Soto J., Llobet E., Brezmes J., Aristoy M.C., Toldráe F. (2008). Fish freshness analysis using metallic potentiometric electrodes. Sens. Actuat B Chem..

[295] Ólafsson R., Martinsdóttir E., Ólafsdóttir G., Sigfússon T.I., Gardner J.W., Gardner J.W., Bartlett P.N. (1992). Monitoring of fish freshness using tin oxide sensors. Sensors and Sensory Systems for an Electronic Nose.

[296] Schweizer-Berberich P.M., Vaihinger S., Gopel W. (1994). Characterisation of food freshness with sensor arrays. Sens. Actuator or B Chem..

[297] Davide F.A.M., di Natale C., D'Amico A. (1995). Self-organizing sensory maps in odour classification mimicking. Biosens. Bioelectron..

[298] Di Natale C., Davide F.A.M., D'Amico A., Sberveglieri G., Nelli P., Faglia G., Perego C. (1995). Complex chemical pattern recognition with sensor array: The discrimination of vintage years of wine. Sens. Actuat or B Chem..

[299] Di Natale C., Brunink J.A.J., Bungaro F. (1996). Recognition of fish storage time by a metalloporphorins-coated QMB sensor array. Meas. Sci. Technol..

[300] Egashira M. Functional design of semiconductor gas sensors for measurement of smell and freshness.

[301] Du W.-X., Lin C.-M., Huang T., Kim J., Marshall M., Wei C.-I. (2002). Potential application of the electronic nose for quality assessment of salmon filets under various storage conditions. J. Food Sci..

[302] Zhang H., Wang J., Tian X., Yu H., Yu Y. (2007). Optimization of sensor array and detection of stored duration of wheat by electronic nose. J. Food Eng..

[303] Limbo S., Sinelli N., Torri L., Riva M. (2009). Freshness decay and shelf life predictive modelling of European sea bass (*Dicentrarchus labrax*) applying chemical methods and electronic nose. LWT Food Sci. Technol..

[304] Barbri N.E., Mirhisse J., Ionescu R., Bari N.E., Correig X., Bouchikhi B., Llobet E. (2009). An electronic nose system based on a micro-machined gas sensor array to assess the freshness of sardines. Sens. Actuat or B Chem..

[305] Musatov V.Y., Sysoev V.V., Sommer M., Kiselev I. (2010). Assessment of meat freshness with oxide sensor microarray electronic nose: A practical approach. Sens. Actuat or B Chem..

[306] Tian X.-Y., Cai Q., Zhang Y.-M. (2012). Rapid classification of hairtail fish and pork freshness using an electronic nose based on the PCA method. Sensors.

[307] Ampuero S., Zesiger T., Gustafsson V., Lunden A., Bosset J.O. (2002). Determination of trimethylamine in milk using an MS based electronic nose. Eur. Food Res. Technol..

[308] Campagnoli A., Pinotti L., Tognon G., Cheli F., Baldi A., Dell'Orto V. (2004). Potential application of electonic nose in processed animal proteins (PAP) detection in feedstuffs. Biotechnol. Agron. Soc..

[309] Logrieco A., Arrigan D.W.M., Brengel-Pesce K., Siciliano P., Tothill I. (2005). DNA arrays, electronic noses and tongues, biosensors and receptors for rapid detection of toxigenic fungi and mycotoxins: A review. Food Addit. Contam..

[310] Zhang S., Xie C., Bai Z., Hu M., Li H., Zeng D. (2009). Spoiling and formaldehyde-containing detections in octopus with an E-nose. Food Chem..

[311] Chobtang J., de Boer I.J.M., Hoogenboom R.L.A.P., Haasnoot W., Kijlstra A., Meerburg B.G. (2011). The need and potential of biosensors to detect dioxins and dioxin-like polychlorinated biphenyls along the milk, eggs, and meat food chain. Sensors.

[312] Baranauskien R., Bylait E., Ukauskait J., Venskutonis R.P. (2007). Flavor retention of peppermint (*Mentha piperita* L.) essential oil spray dried in modified starches during encapsulation and storage. J. Agric. Food Chem..

[313] Köster E.P. (2005). Does olfactory memory depend on remembering odors?. Chem. Sens..

[314] Köster M.A., Prescott J., Köster E.P. (2004). Incidental learning and memory for three basic tastes in food. Chem. Sens..

[315] Le Berre E., Thomas-Danguin T., Béno N., Coureaud G., Etiévant P., Prescott J. (2008). Perceptual processing strategy and exposure influence the perception of odor mixtures. Chem. Sens..

[316] Murphy C., Cain W.S., Bartoshuk L.M. (1977). Mutual action of taste and olfaction. Sens. Process..

[317] Murphy C., Cain W.S. (1980). Taste and olfaction: independence *vs.* interaction. Physiol. Behav..

[318] Auvray M., Spence C. (2008). The multisensory perception of flavor. Conscious. Cogn..

[319] Lee S., Park T. (2010). Recent advances in the development of bioelectronic nose. Biotechnol. Bioproc. Eng..

[320] Baldwin E.A., Bai J., Plotto A., Dea S. (2011). Electronic noses and tongues: Application for the food and pharmaceutical industries. Sensors.

[321] Davis T.W., Kuo C.-M., Liang X., Yu P.-S. (2012). Sap flow sensors: Construction, quality control and comparison. Sensors.

[322] Contreras-Medina L.M., Osornio-Rios R.A., Torres-Pacheco I., Romero-Troncoso R., Guevara-González R.G., Millan-Almaraz J.R. (2012). Smart sensor for real-time quantification of common symptoms present in unhealthy plants. Sensors.

[323] Baietto M., Wilson A.D. (2010). Relative *in vitro* wood decay resistance of sapwood from landscape trees of southern temperate regions. Hort. Sci..

[324] Garneau F.X., Riedl B., Hobbs S., Pichette A., Gagnon H. (2004). The use of sensor array technology for rapid differentiation of the sapwood and heartwood of Eastern Canadian spruce; fir and pine. Holz Roh Werkst..

[325] Murphy G., Franich R. (2004). Early experience with aroma tagging and electronic nose technology for log tracking. For. Prod. J..

[326] Murphy G. Early experience with aroma tagging and electronic nose technology for log and forest products tracking.

[327] Zheng Y., Liu J., Wang D., Yang R. (2012). Laser scanning measurements on trees for logging harvesting operations. Sensors.

[328] Baby R.E., Cabezas M., Walsoe de Reca E.N. (2000). Electronic nose: A useful tool for monitoring environmental contamination. Sens. Actuator B Chem..

[329] Canhoto O., Magan N. (2003). Potential for the detection of microorganisms and heavy metals in potable water using electronic nose technology. Biosens. Bioelectron..

[330] Goschnick J., Koronczi I., Frietsch M., Kiselev I. (2005). Water pollution recognition with the electronic nose KAMINA. Sens. Actuator B Chem..

[331] Bourgeois. W., Stuetz R.M. (2002). Use of a chemical sensor array for detecting pollutants in domestic wastewater. Water Res..

[332] Lamagna A., Reich S., Rodríquez D., Boselli A., Cicerone D. (2008). The use of an electronic nose to characterize emissions from a highly polluted river. Sens. Actuator B Chem..

[333] Falasconi M., Gobbi E., Pardo M., Torre M.D., Bresciani A., Sberveglieri G. (2005). Detection of toxigenic strains of *Fusarium verticillioides* in corn by electronic olfactory system. Sens. Actuator B Chem..

[334] Cheli F., Campagnoli A., Pinotti L., Savoini G., Dell'Orto V. (2009). Electronic nose for determination of aflatoxins in maize. Biotechnol. Agron. Soc..

[335] Campagnoli A., Cheli F., Savoini G., Crotti A., Pastori A.G.M., Dell'Orto V. (2009). Application of an electronic nose to detection of aflatoxins in corn. Vet. Res. Commun..

[336] Tang K., Chiu S., Pan C., Hsieh H., Liang Y., Liu S. (2010). Development of a portable electronic nose system for the detection and classification of fruity odors. Sensors.

[337] Cimander C., Mandenius C. (2002). Online monitoring of a bioprocess based on a multi-analyser system and multivariate statistical process modeling. J. Chem. Technol. Biotechnol..

[338] Cimander C., Carlsson M., Mandenius C. (2002). Sensor fusion for on-line monitoring of yoghurt fermentation. J. Biotechnol..

[339] Markoma M.A., Md Shakaff A.Y., Adom A.H., Ahmad M.N., Hidayat W., Abdullah A.H., Fikri N.A. (2009). Intelligent electronic nose system for basal stem rot disease detection. Comput. Electron. Agric..

[340] Sironi S., Capelli L., Céntola P., Del Rosso R., Grande M.I. (2007). Continuous monitoring of odours from a composting plant using electronic noses. Waste Manag..

[341] Littarru P. (2007). Environmental odours assessment from waste treatment plants: Dynamic olfactometry in combination with sensorial analysers “electronic noses”. Waste Manag..

[342] Maarse H., Visscher C.A. (1996). Volatile Compounds in Foods: Quantitative and Qualitative Data.

[343] Blank I., Sen A., Grosch W. (1992). Potent odorants of the roasted powder and brew of Arabica coffee. Z. Lebensmittelunters. Forsch..

[344] Sarrazin C., Le Quéré J.-L., Gretsch C., Liardon R. (2000). Representativeness of coffee extracts: A comparison of different extraction methods. Food Chem..

[345] Dharmawan J. (2008). Characterization of Volatile Compounds in Selected Citrus Fruits from Asia. Ph.D. Thesis.

[346] Zheng L.-Y., Sun G.-M., Liu Y.-G., Lv L.-L., Yang W.-X., Zhao W.-F., Wei C.-B. (2012). Aroma volatile compounds from two fresh pineapple varieties in China. Int. J. Mol. Sci..

[347] Beaulieu J.C., Lea J.M. (2006). Characterization and semiquantitative analysis of volatiles in seedless watermelon varieties using solid-phase microextraction. J. Agric. Food Chem..

[348] Sáenz C., Cedrón T., Cabredo S. (2010). Classification of wines from five Spanish origin denominations by aromatic compound analysis. J. AOAC Int..

[349] Chang C.-I., Hung P.-H., Wu C.-C., Cheng T.C., Tsai J.-M., Lin K.-J., Lin C.-Y. (2012). Simultaneous detection of multiple fish pathogens using a naked-eye readable DNA microarray. Sensors.

[350] Zhang J., Li P., Hu X., Zhang Q., Ding X., Zhang W. (2012). Microarray technology for major chemical contaminants analysis in food: Current status and prospects. Sensors.

[351] Liu G., Lao R., Xu L., Xu Q., Li L., Zhang M., Shen H., Mathur S., Fan C., Song S. (2011). Detection of single-nucleotide polymorphism on *uidA* gene of *Escherichia coli* by a multiplexed electrochemical DNA biosensor with oligonucleotide-incorporated nonfouling surface. Sensors.

[352] Geschwindner S., Carlsson J.F., Knecht W. (2012). Application of optical biosensors in small-molecule screening activities. Sensors.

[353] Zhang Q., Xue C., Yuan Y., Lee J., Sun D., Xiong J. (2012). Fiber surface modification technology for fiber-optic localized surface plasmon resonance biosensors. Sensors.

[354] Hussain I., Brust M., Papworth A.J., Cooper A.I. (2003). Preparation of acrylate-stabilized gold and silver hydrosols and gold-polymer composite films. Langmuir.

[355] Liu F.-J., Huang L.-M., Wen T.-C., Li C.-F., Huang S.-L., Gopalan A. (2008). Platinum particles dispersed polyaniline-modified electrodes containing sulfonated polyelectrolyte for methanol oxidation. Synth. Met..

[356] O'Mullane A.P., Dale S.E., Macpherson J.V., Unwin P.R. (2004). Fabrication and electrocatalytic properties of polyaniline/Pt nanoparticle composite. Chem.Commun..

[357] Sakmeche N., Bazzaoui E.A., Fall M., Aeiyach S., Jouini M., Lacroix J.C., Aaron J.J., Lacaze P.C. (1997). Application of sodium dodecyl sulphate (SDS) micellar solution as an organised medium for electropolymerization of thiopene derivatives in water. Synth. Met..

[358] Ogura K., Nakaoka K., Nakayama M. (2000). Studies on ion transport during potential cycling of a Prussian blue (inner) polyaniline (outer) bilayer electrode by quartz crystal microbalance and Fourier transform infrared reflection spectroscopy. J. Electroanal. Chem..

[359] Lupu S., Mihailiciuc C., Pigani L., Renato S., Nicolae T., Chiara Z. (2002). Electrochemical preparation and characterization of bilayer film composed by Prussian blue and conducting polymer. Electrochem. Commun..

[360] Schilirò T., Pignata C., Rovere R., Fea E., Gilli G. (2009). The endocrine disrupting activity of surface waters and of wastewater treatment plant effluents in relation to chlorination. Chemosphere.

[361] Olowu R.A., Arotiba O., Mailu S.N., Waryo T.T., Baker P., Iwuoha E. (2010). Electrochemical aptasensor for endocrine disrupting 17β-Estradiol based on a poly(3,4-ethylenedioxylthiopene)-Gold nanocomposite platform. Sensors.

[362] Gallardo J., Alegret S., Muñoz R., Leija L., Hernandez P.R., del Valle M. (2003). An electronic tongue using potentiometric all-solid-state PVC-membrane sensors for the simultaneous quantification of ammonium and potassium ions in water. Anal. Bioanal. Chem..

[363] Gallardo J., Alegret S., Muñoz R., Leija L., Hernandez P.R., del Valle M. (2005). Use of an electronic tongue based on all-solidstate potentiometric sensors for the quantitation of alkaline ions. Electroanalysis.

[364] Gutierrez M., Alegret S., del Valle M. (2007). Potentiometric bioelectronic tongue for the analysis of urea and alkaline ions in clinical samples. Biosens. Bioelectron..

[365] Cortina M., Duran A., Alegret S., del Valle M. (2006). A sequential injection electronic tongue employing the transient response from potentiometric sensors for anion multidetermination. Anal. Bioanal. Chem..

[366] Gutes A., Calvo D., Cespedes F., del Valle M. (2007). Automatic sequential injection analysis electronic tongue with integrated reference electrode for the determination of ascorbic acid, uric acid and paracetamol. Microchim. Acta.

[367] Krantz-Rülcker C., Stenberg M., Winquist F., Lundström I. (2001). Electronic tongues for environmental monitoring based on sensor arrays and pattern recognition: A review. Anal. Chim. Acta.

[368] Vlasov Y., Legin A., Rudnitskaya A. (1997). Cross-sensitivity evaluation of chemical sensors for electronic tongue: determination of heavy metal ions. Sens. Actuator B Chem..

[369] Gallardo J., Alegret S., del Valle M. (2004). A flow-injection electronic tongue based on potentiometric sensors for the determination of nitrate in the presence of chloride. Sens. Actuator B Chem..

[370] Gutes A., Cespedes F., Alegret S., del Valle M. (2005). Sequential injection system with higher dimensional electrochemical sensor signals: Part 1. Voltammetric e-tongue for the determination of oxidizable compounds. Talanta.

[371] Gutes A., Cespedes F., del Valle M., Louthander D., Krantz-Rülcker C., Winquist F. (2006). A flow injection voltammetric electronic tongue applied to paper mill industrial waters. Sens. Actuator B Chem..

[372] Valdes-Ramírez G., Gutierrez M., del Valle M., Ramírez-Silva M.T.D., Fournier D., Marty J.L. (2009). Automated resolution of dichlorvos and methylparaoxon pesticide mixtures employing a flow injection system with an inhibition electronic tongue. Biosens. Bioelectron..

[373] Gutes A., Ibañez A.B., Cespedes F., Alegret S., del Valle M. (2005). Simultaneous determination of phenolic compounds by means of an automated voltammetric “electronic tongue”. Anal. Bioanal. Chem..

[374] Gutierrez M., Alegret S., Caceres R., Casadesús J., Marf O., del Valle M. (2007). Application of a potentiometric electronic tongue to fertigation strategy in greenhouse cultivation. Comput. Electron. Agric..

[375] Vlasov Y., Legin A., Rudnitskaya A., di Natale C., D'amico A. (2005). Nonspecific sensor arrays (“electronic tongue”) for chemical analysis of liquids. Pure Appl. Chem..

[376] Escuder-Gilabert L., Peris M. (2010). Review: Highlights in recent applications of electronic tongues in food analysis. Anal. Chim. Acta.

[377] Riul A., Dantas C.A.R., Miyazaki C.M., Oliveira O.N. (2010). Recent advances in electronic tongues. Analyst.

[378] del Valle M. (2012). Sensor arrays and electronic tongue systems. Int. J. Electrochem..

[379] Guiseppi-Elie A. (2010). Electroconductive hydrogels: Synthesis, characterization and biomedical applications. Biomaterials.

[380] Park S., Lee H.J., Koh W.-G. (2012). Multiplex immunoassay platforms based on shape-coded poly(ethylene glycol) hydrogel microparticles incorporating acrylic acid. Sensors.

[381] Spinelli F., Noferini M., Costa G. (2006). Near infrared spectroscopy (Nirs): Perspective of fire blight detection in asymptomatic plant material. Acta Hort..

[382] Sankaran S., Mishra A., Ehsani R., Davis C. (2010). A review of advanced techniques for detecting plant diseases. Comput. Electron. Agric..

[383] Brudzewski K., Osowski S., Ulaczyk J. (2010). Differential electronic nose of two chemo sensor arrays for odor discrimination. Sens. Actuator B Chem..

[384] Choi S.-I., Kim S.-H., Yang Y., Jeong G.-M. (2010). Data refinement and channel selection for a portable e-nose system by the use of feature feedback. Sensors.

[385] Twomey K., de Eulate E.A., Alderman J., Arrigan D.W.M. (2009). Fabrication and characterization of a miniaturized planar voltammetric sensor array for use in an electronic tongue. Sens. Actuator B Chem..

[386] Sun Y.-F., Liu S.-B., Meng F.-L., Liu J.-Y., Jin Z., Kong L.-T., Liu J.-H. (2012). Metal oxide nanostructures and their gas sensing properties: A review. Sensors.

[387] Chen P.-C., Shen G., Zhou C. (2008). Chemical sensors and electronic noses based on 1-D metal oxide nanostructures. IEEE Trans. Nanotechnol..

[388] García-González D.L., Aparicio R. (2002). Sensors: From biosensors to the electronic nose. Grasas y Aceites.

[389] Staples E.J. Electronic nose simulation of olfactory response containing 500 orthogonal sensors in 10 seconds.

[390] White J., Kauer J.S., Dickinson T.A., Walt D.R. (1996). Rapid analyte recognition in a device based on optical sensors and the olfactory system. Anal. Chem..

[391] Campagnoli A., Cheli F., Polidori C., Zaninelli M., Zecca O., Savoini G., Pinotti L., Dell'Orto V. (2011). Use of the electronic nose as a screening tool for the recognition of durum wheat naturally contaminated by deoxynivalenol: A preliminary approach. Sensors.

[392] Fujioka K., Arakawa E., Kita J., Aoyama Y., Okuda T., Yoshinobu M., Yamamoto K. (2009). Combination of real-value smell and metaphor expression aids yeast detection. PLoS One.

[393] Falasconi M., Concina I., Gobbi E., Sberveglieri V., Pulvirenti A., Sberveglieri G. (2012). Electronic nose for microbiological quality control of food products. Int. J. Electrochem..

[394] Wang D., Wang X., Liu T., Liu Y. (2012). Prediction of total viable counts on chilled pork using an electronic nose combined with support vector machine. Meat Sci..

[395] Pallottino F., Costa C., Antonucci F., Strano M.C., Calandra M., Solainia S., Menesattia P. (2012). Electronic nose application for determination of *Penicillium digitatum* in Valencia oranges. J. Sci. Food Agric..

[396] Gobbi E., Falasconi M., Torelli E., Sberveglieri G. (2011). Electronic nose predicts high and low fumonisin contamination in maize cultures. Food Res. Int..

[397] Zhang B., Xi W.P., Wei W.W., Shen J.Y., Ferguson I., Chen K.S. (2011). Changes in aroma-related volatiles and gene expression during low temperature storage and subsequent shelf-life of peach fruit. Postharvest Biol. Tecnol..

[398] Zhang X., Qi Y., Yang X., Jia H. (2012). Evaluation of maturity of peach by electronic nose. J. South China Agric. Univ..

[399] Olunloyo V.O.S., Ibidapo T.A., Dinrifo R.R. (2011). Neural network-based electronic nose for cocoa beans quality assessment. Agric. Eng. Int. CIGR J..

[400] Messina V., Domínguez P.G., Sancho A.M., Walsöe de Reca N., Carrari F., Grigioni G. (2012). Tomato quality during short-term storage assessed by colour and electronic nose. Int. J. Electrochem..

[401] Romani S., Cevoli C., Fabbri A., Alessandrini L., Rosa M.D. (2012). Evaluation of coffee roasting degree by using electronic nose and artificial neural network for off-line quality control. J. Food Sci..

[402] Kubiak A., Wenzl T., Ulberth F. (2012). Evaluation of the quality of postharvest rapeseed by means of an electronic nose. J. Sci. Food Agric..

[403] Yang Y., Zhao Y., Zhang S., Ni Y., Zhan J. (2012). Qualitative analysis of age and brand of unblended brandy by electronic nose. Computer and Computing Technologies in Agriculture V, IFIP Advances in Information and Communication Technology.

[404] Berna A. (2010). Metal oxide sensors for electronic noses and their application to food analysis. Sensors.

[405] Vallone S., Lloyd N.W., Ebeler S.E., Zakharov F. (2012). Fruit volatile analysis using an electronic nose. J. Vis. Exp..

[406] Demir N., Ferraz A.C., Sargent S.A., Balaban M.O. (2011). Classification of impacted blueberries during storage using an electronic nose. J. Sci. Food Agric..

[407] Mamat M., Samad S.A., Hannan M.A. (2011). An electronic nose for reliable measurement and correct classification of beverages. Sensors.

[408] Ghasemi-Varnamkhasti M., Mohtasebi S.S., Siadat M., Razavi S.H., Ahmadi H., Dicko A. (2012). Discriminatory power assessment of the sensor array of an electronic nose system for the detection of non-alcoholic beer aging. Czech J. Food Sci..

[409] Ghaffari R., Zhang F., Iliescu D., Hines E., Leeson M., Napier R., Clarkson J. Early detection of diseases in tomato crops: An electronic nose and intelligent systems approach.

[410] Lee W.S., Alchanatis V., Yang C., Hirafuji M., Moshoue D., Li C. (2010). Sensing technologies for precision specialty crop production. Comput. Electron. Agric..

[411] Zhou B., Wang J. (2011). Detection of insect infestations in paddy field using an electronic nose. Int. J. Agric. Biol..

[412] Li C., Schmidt N.E., Gitaitis R. (2011). Detection of onion postharvest diseases by analyses of headspace volatiles using a gas sensor array and GC-MS. Food Sci. Technol..

[413] Abdullah A.H., Adom A.H., Shakaff A.Y.M., Ahmad M.N., Zakaria A., Saad F.S.A., Isa C.M.N.C., Masnan M.J., Kamarudin L.M. Hand-held electronic nose sensor selection system for basal stamp rot (BSR) disease detection.

[414] Fogg S. Electronic nose could be integrated into smartphones. http://www.newelectronics.co.uk/electronics-news/lectronic-nose-could-be-integrated-into-smartphones/44415/.

[415] Kinkeldei T., Zysset C., Münzenrieder N., Petti L., Tröster G. (2012). In tube integrated electronic nose system on a flexible polymer substrate. Sensors.

[416] Santos J.P., Aleixandrea M., Cruz C. (2012). Hand held electronic nose for VOC detection. Chem. Eng. Trans..

[417] Hamedani N.F., Mahjoub A.R., Ali khodadadi A., Mortazavi Y. CO and ethanol selective sensor of La_2_O_3_-doped ZnO nanostructures synthesized by microwave assisted fast method.

[418] Amin M., Manzoor U., Islam M., Bhatti A.S., Shah N.A. (2012). Synthesis of ZnO nanostructures for low temperature CO and UV sensing. Sensors.

[419] Mirabbaszadeh K., Mehrabian M. (2012). Synthesis and properties of ZnO nanorods as ethanol gas sensors. Phys. Scr..

[420] Tian X.Y., Cai Q., Ye Z.X., Guo W., Lu Y.W., Zhang Y.M. (2011). Detection of TVOC and odor in industrial park using electronic nose. Huan Jing Ke Xue.

[421] Oladipupo O.O., Eletta O.A. (2012). Neuro-identification of some commonly used volatile organic compounds using electronic nose. Chem. Process Eng. Res..

[422] Kim H., Konnanath B., Sattigeri P., Wang J., Mulchandani A., Myung N., Deshusses M.A., Spanias A., Bakkaloglu B. (2012). Electronic-nose for detecting environmental pollutants: Signal processing and analog front-end design. Analog Integr. Circ. Signal Process..

[423] Pan L., Yang S.X. (2009). An electronic nose network system for online monitoring of livestock farm odors. IEEE Trans. Mechatron..

[424] Brattoli M., de Gennaro G., de Pinto V., Loiotile A.D., Lovascio S., Penza M. (2011). Odour detection methods: Olfactometry and chemical sensors. Sensors.

[425] Abdullah A.H., Shakaff A.Y.M., Adom A.H., Zakaria A., Saad F.S.A., Kamarudin L.M. (2012). Chicken farm malodour monitoring using portable electronic nose system. Chem. Eng. Trans..

[426] Dentoni L., Capelli L., Sironi S., Rosso R.D., Zanetti S., Torre M.D. (2012). Development of an electronic nose for environmental odour monitoring. Sensors.

[427] Lan J., Liu B., Chen Z., Song Z., Lin J. (2012). Discriminate model of electronic nose for distinguishing volatiles of microbial fermentation bed in swine house. Fujian J. Agric. Sci..

[428] Zhang M., Wang X., Liu Y., Xu X., Zhou G. (2012). Species discrimination among three kinds of puffer fish using an electronic nose combined with olfactory sensory evaluation. Sensors.

[429] De Cesare F., di Mattia E., Pantalei S., Zampetti E., Vinciguerra V., Macagnano A. (2011). Electronic nose technology to measure soil microbial activity and classify soil metabolic status. Nat. Precedings.

[430] Zhang F., Iliescu D.D., Hines E.L., Leeson M.S., Hines E.L., Leeson M.S. (2011). Tomato plant health monitoring: An electronic nose approach. Intelligent Systems for Machine Olfaction: Tools and Methods.

